# GLP-1 release and vagal afferent activation mediate the beneficial metabolic and chronotherapeutic effects of D-allulose

**DOI:** 10.1038/s41467-017-02488-y

**Published:** 2018-01-09

**Authors:** Yusaku Iwasaki, Mio Sendo, Katsuya Dezaki, Tohru Hira, Takehiro Sato, Masanori Nakata, Chayon Goswami, Ryohei Aoki, Takeshi Arai, Parmila Kumari, Masaki Hayakawa, Chiaki Masuda, Takashi Okada, Hiroshi Hara, Daniel J. Drucker, Yuichiro Yamada, Masaaki Tokuda, Toshihiko Yada

**Affiliations:** 1 Division of Integrative Physiology, Department of Physiology, Jichi Medical University School of Medicine, 3311-1 Yakushiji, Shimotsuke, Tochigi 329-0498 Japan; 2 Research Faculty of Agriculture, Hokkaido University, Kita-9, Nishi-9, Kita-ku, Sapporo 060-8589 Japan; 3 Department of Endocrinology, Diabetes and Geriatric Medicine, Akita University Graduate School of Medicine, 1-1-1 Hondo, Akita, 010-8543 Japan; 4 Graduate School of Agriculture, Hokkaido University, Kita-9, Nishi-9, Kita-ku, Sapporo 060-8589 Japan; 5 Department of Biochemistry and Molecular Biology, Nippon Medical School, 1-1-5 Sendagi, Bunkyo-ku, Tokyo 113-8602 Japan; 6 Lunenfeld Tanenbaum Research Institute, Mt. Sinai Hospital, 600 University Avenue TCP5-1004 Mailbox 39, Toronto, ON M5G 1X5 Canada; 7 Faculty of Medicine, Department of Cell Physiology, Kagawa University, 1750-1, Ikenobe, Miki-cho, Kita-gun, Kagawa 761-0793 Japan; 8 Kansai Electric Power Medical Research Institute, 1-5-6 Minatojimaminamimachi, Chuou-ku, Kobe 650-0047 Japan

## Abstract

Overeating and arrhythmic feeding promote obesity and diabetes. Glucagon-like peptide-1 receptor (GLP-1R) agonists are effective anti-obesity drugs but their use is limited by side effects. Here we show that oral administration of the non-calorie sweetener, rare sugar d-allulose (d-psicose), induces GLP-1 release, activates vagal afferent signaling, reduces food intake and promotes glucose tolerance in healthy and obese-diabetic animal models. Subchronic d-allulose administered at the light period (LP) onset ameliorates LP-specific hyperphagia, visceral obesity, and glucose intolerance. These effects are blunted by vagotomy or pharmacological GLP-1R blockade, and by genetic inactivation of GLP-1R signaling in whole body or selectively in vagal afferents. Our results identify d-allulose as prominent GLP-1 releaser that acts via vagal afferents to restrict feeding and hyperglycemia. Furthermore, when administered in a time-specific manner, chronic d-allulose corrects arrhythmic overeating, obesity and diabetes, suggesting that chronotherapeutic modulation of vagal afferent GLP-1R signaling may aid in treating metabolic disorders.

## Abstract (web summary)

The sweetener D-allulose has beneficial metabolic effects in animal models, but its mechanism of action was unclear. Here the authors report that D-allulose triggers GLP-1 release in the gut and GLP-1R signaling on vagal afferents, counteracting arrhythmic overeating, obesity and diabetes.

## Introduction

The obesity pandemic is a serious worldwide health problem: 39% of adults (1.9 billion) are overweight/obesity^1^, and these numbers are increasing^2^. Obesity is a major risk factor for type 2 diabetes, cardiovascular, and cerebral diseases. Overeating and abnormal feeding rhythm contribute to development of obesity^3,4^. However, few effective and safe medicines and/or food ingredients (supplements) are available to treat obesity and feeding disorders. One exception is the class of glucagon-like peptide-1 receptor (GLP-1R) agonists, which can be used to treat not only type 2 diabetes but obesity, hyperphagia, and hypertension^5–7^. Recent randomized double-blind trials showed that treatment with GLP-1R agonists reduces cardiovascular events, stroke, and nephropathy^8,9^. However, GLP-1R agonists elicit nausea and vomiting^6,10^, and increase mean heart rate via directly stimulating sinoatrial node and enhancing sympathetic nervous system tone^10–12^. The mechanisms underlying these adverse effects of GLP-1R agonists remain to be elucidated. However, their long life-span, penetration through the blood–brain barrier (BBB)^13^, and high therapeutic doses may allow them to directly act on central nervous system GLP-1Rs^12^, thereby causing CNS-dependent side effects. Strategies to enhance the tolerability of GLP-1-based therapy might include identification of agents that stimulate release of endogenous GLP-1 from intestinal L-cells, potentially accompanied by fewer adverse effects.

d-Allulose (d-*ribo*-2-hexylose, previously named d-psicose), a C-3 epimer of d-fructose, is a rare sugar existing in small amounts in nature. d-Allulose contributes no calories but substantial sweetness equivalent to glucose and 70% of sucrose^14,15^, yielding a zero-calorie sweetener. In humans, large proportions of orally administered d-allulose are absorbed and subsequently excreted via the urine without being metabolized^14^. d-Allulose has been shown to ameliorate diabetes and obesity in animals^16–18^, however, underlying mechanisms are largely unclear. Of particular importance is whether d-allulose alters feeding, a behavior upstream of glucose and energy homeostasis.

The previous reports of multiple beneficial effects of orally administered d-allulose prompted us to infer that it might act through stimulating GLP-1 release and/or vagal afferent signaling, both of which transmit gut signals to remote organs including the brain. Notably, GLP-1 directly interacts with vagal afferent neurons^19^, and surgical or chemical denervation of vagal afferents and specific *Glp1r* knockdown in vagal afferent neurons attenuate anorexigenic, insulinotropic and glycemic effects of exogenous GLP-1 and dipeptidyl peptide-4 (DPP-4; an enzyme that degrades GLP-1) inhibitors^20–25^. Thus, although it is evident that vagal afferents play a key role in metabolic effects of GLP-1, it is not clear whether d-allulose exerts its metabolic actions through GLP-1 and vagal afferent signaling.

We examined the effects of d-allulose on feeding behavior including arrhythmic hyperphagia that is characteristically associated with obesity and diabetes^3,4^ and on glucose metabolism by glucose tolerance test (GTT), insulin tolerance test (ITT), and pyruvate tolerance test (PTT), and explored underlying mechanisms involving GLP-1 release and vagal afferents using *Glp1r* knockout mice (*Glp1r* KO), surgical vagotomy and *Glp1r* knockdown specifically in vagal afferents using viral-mediated shRNA. We found that oral administration of d-allulose induces GLP-1 release, activates vagal afferents, reduces food intake, and promotes glucose tolerance via enhanced insulin secretion and action. Subchronic administration of d-allulose ameliorates arrhythmic hyperphagia, obesity, and glucose intolerance. These effects are blunted by vagotomy and by pharmacological or genetic inactivation of the GLP-1R in vagal afferents. Our results demonstrate that d-allulose stimulates GLP-1 release, and corrects arrhythmic overeating, obesity and diabetes via vagal afferent pathways.

## Results

### Peroral d-allulose suppresses food intake without aversion

**Fig. 1:**
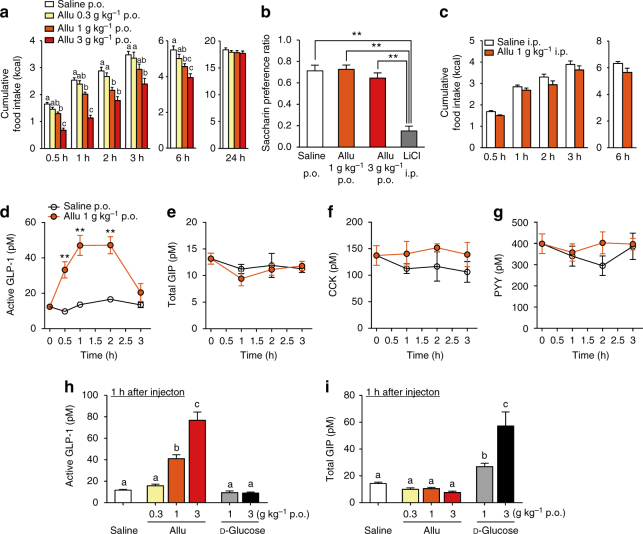
Peroral d-allulose suppresses food intake and releases GLP-1 in normal mice. **a** Cumulative food intake at 0.5–24 h after p.o. administration of 0.3, 1, and 3 g kg^−1^ d-allulose (Allu) in C57BL/6J mice fasted overnight (16 h). *n* = 10. Different letters indicate *p* < 0.05 by one-way ANOVA followed by Tukey’s test in each time. **b** In conditioned taste aversion test, saccharin preference was measured at 2 days after injection of saline, Allu, or lithium chloride (LiCl; 3 mmol kg^−1^). *n* = 5–8. **p < 0.01 by one-way ANOVA followed by Tukey’s test. **c** Cumulative food intake after intraperitoneal (i.p.) injection of 1 g kg^−1^ Allu. *n* = 8. **d**–**g** Time course of active GLP-1 (**d**), total GIP (**e**), CCK (**f**), and PYY (**g**) concentrations in portal vein plasma after 1 g kg^−1^ Allu or saline p.o. injection. *n* = 5–9. **p < 0.01 by two-way ANOVA followed by Bonferroni’s test vs. saline. **h**, **i** Active GLP-1 (**h**) and total GIP (**i**) concentrations in portal vein at 1 h after p.o. administration of increasing concentrations of Allu or d-glucose. *n* = 5–15. Different letters indicate *p* < 0.05 by one-way ANOVA followed by Tukey’s test. Error bars are SEM

Peroral (p.o.) administration of d-allulose (Allu) at 1 and 3 g kg^−1^, but not 0.3 g kg^−1^, into the stomach using a stainless feeding needle decreased cumulative food intake for 0.5, 1, 2, 3, and 6 h after injection in C57BL/6J mice fasted overnight (16 h) (Fig. 1a). Subsequently, cumulative food intake at 24 h after p.o. d-allulose returned to normal levels and body weight was not altered at 24 h (Fig. 1a and Supplementary Fig. 2a, b). In contrast, d-glucose, p.o. administered at 1 and 3 g kg^−1^, did not alter food intake at any time point (Supplementary Fig. 1). d-Allulose (1 and 3 g kg^−1^) did not induce taste aversion, unlike lithium chloride (Fig. 1b). Moreover, ingestion of d-allulose suppressed food intake without altering water intake, urine volume and osmolality, creatinine excretion, glucose excretion, and electrolytes (Na^+^, K^+^, and Cl^−^) excretion (Supplementary Fig. 2). Food intake was not significantly altered by intraperitoneal (i.p.) injection of d-allulose at 1 g kg^−1^ (Fig. 1c) and at 3 g kg^−1^ (saline i.p. vs. d-allulose 3 g kg^−1^ i.p.; 4.03 ± 0.12 vs. 3.46 ± 0.37 kcal at 3 h, 6.20 ± 0.14 vs. 5.56 ± 0.24 kcal at 6 h, *n* = 8–14, not significant by unpaired *t*-test). Thus, oral, but not i.p., administration of d-allulose suppressed food intake without inducing aversive behavior and influencing kidney functions.

### Oral administration of d-allulose induces GLP-1 secretion

As oral administration of d-allulose was particularly effective, we explored the involvement of gut-related mechanisms. Active GLP-1 concentrations in portal vein significantly increased at 0.5 h and plateaued at 1 and 2 h after oral administration of d-allulose (1 g kg^−1^), returning to baseline at 3 h (Fig. 1d). In contrast, total glucose-dependent insulinotropic polypeptide (GIP), cholecystokinin (CCK), and peptide YY (PYY) concentrations did not change (Fig. 1e–g). At 1 h after injection, d-allulose at 1 and 3 g kg^−1^, but not 0.3 g kg^−1^, dose dependently increased portal active GLP-1 levels (Fig. 1h), without influencing GIP levels (Fig. 1i). In contrast, d-glucose (1 and 3 g kg^−1^ p.o.) increased total GIP concentrations, without influencing GLP-1 levels (Fig. 1h, i). The dose dependency and time course of the effects of d-allulose on GLP-1 release were correlated with those on food intake (Fig. 1d, h vs. Fig. 1a).

### d-Allulose decreases food intake via GLP-1R signaling

**Fig. 2:**
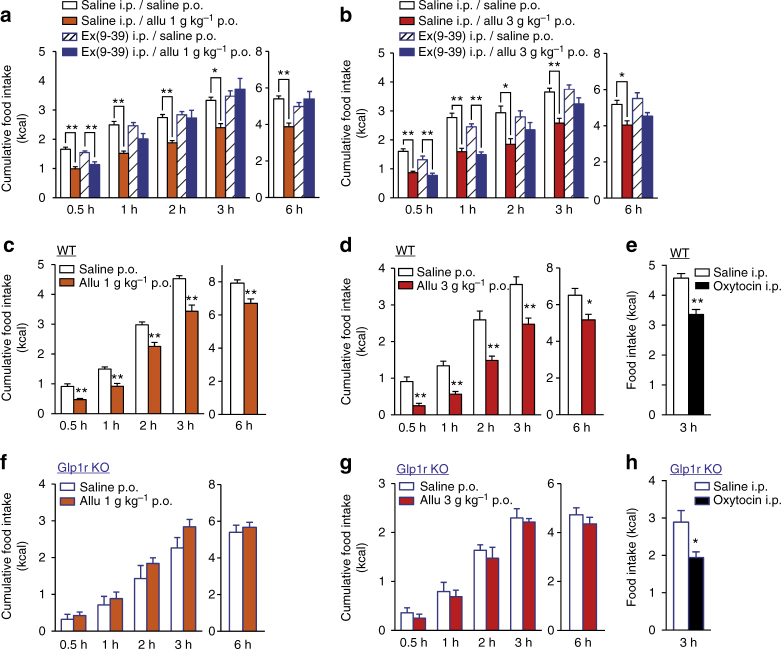
GLP-1 receptor is essential for d-allulose-induced anorexigenic effect. **a**, **b** p.o. Allu at 1 g kg^−1^ (**a**) and 3 g kg^−1^ (**b**) at 10:00 suppressed cumulative food intake at 0.5–6 h after administration in C57BL/6J mice fasted overnight. These anorexigenic effects were attenuated by 200 nmol kg^−1^ Ex(9-39), GLP-1 receptor (GLP-1R) antagonist. *n* = 5–9. *p < 0.05 and **p < 0.01 by one-way ANOVA followed by Tukey’s test. **c**–**h** Cumulative food intake at 0.5–6 h after p.o. injection of 1 and 3 g kg^−1^ Allu and i.p. injection of 400 nmol kg^−1^ oxytocin at 19:30 in wild-type (WT) C57BL/6J mice (**c**–**e**) and *Glp1r* KO mice (**f**–**h**) fasted 3 h. *n* = 5–8. *p < 0.05 and **p < 0.01 by unpaired *t*-test. Error bars are SEM

To assess whether GLP-1 mediates d-allulose-induced inhibition of food intake we used a GLP-1R antagonist, exendin(9-39) (Ex(9-39)). In the presence of 200 nmol kg^−1^ Ex(9-39), the action of p.o. 1 g kg^−1^ d-allulose to suppress food intake was not significantly altered at 0.5 h, but the suppression of food intake was mildly attenuated at 1 h and blunted at 2, 3, and 6 h (Fig. 2a). The action of higher dose (3 g kg^−1^) of d-allulose to suppress food intake was not significantly altered by Ex(9-39) at 0.5 and 1 h, but markedly attenuated at 2, 3, and 6 h (Fig. 2b).

To further explore the involvement of GLP-1R signaling in the anorexigenic effect of d-allulose, we used *Glp1r* knockout (*Glp1r* KO) mice^26^. Oral d-allulose at 1 and 3 g kg^−1^ failed to alter food intake at any time point from 0.5 to 6 h in *Glp1r* KO mice (Fig. 2f, g), while it inhibited food intake at all time points in control wild-type (WT) C57BL/6J mice (Fig. 2c, d). I.p. administration of oxytocin, which inhibits food intake via activation of vagal afferents^27^, exerted its anorexigenic effect in *Glp1r* KO and WT mice to similar extents (Fig. 2e, h). These results indicate that GLP-1R signaling is required for the anorexigenic effect of d-allulose.

### d-Allulose improves glucose tolerance via GLP-1R signaling

**Fig. 3:**
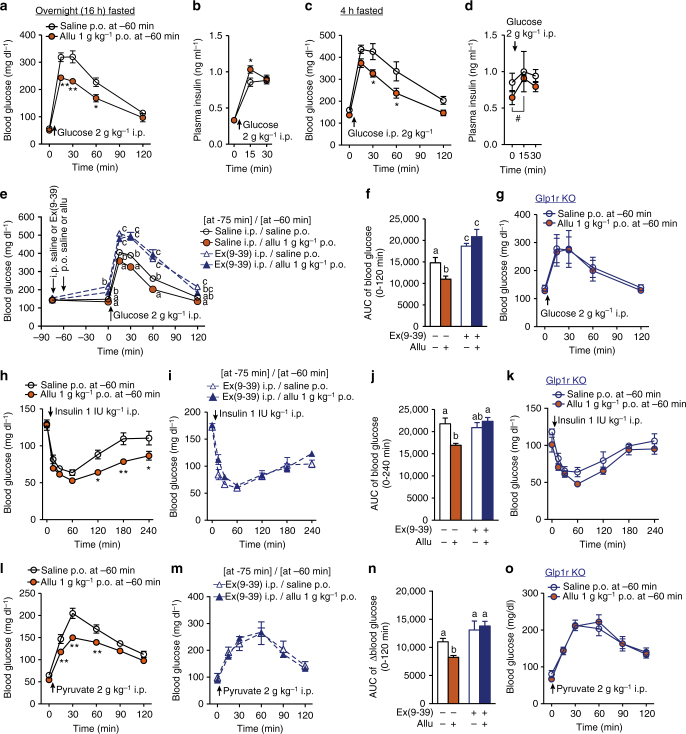
d-Allulose improves glucose tolerance via GLP-1R signaling. Allu at 1 g kg^−1^ was p.o. administered at 60 min prior to ipGTT (2 g kg^−1^), **a**–**g**, insulin tolerance test (ITT, 1 IU kg^−1^, **h**–**k**) and pyruvate tolerance test (PTT, 2 g kg^−1^, **l-o**). Ex(9-39) at 200 nmol kg^−1^ was i.p. injected at 75 min prior to ipGTT (**e**, **f**), ITT (**i**, **j**), and PTT (**m**, **n**). **a**–**d** Effect of p.o. Allu on blood glucose and plasma insulin levels in ipGTT in C57BL/6J mice fasted overnight (16 h, **a**, **b**) and for 4 h (**c**, **d**). *n* = 5. **e**, **f** Ex(9-39) treatment blunted the action of Allu to attenuate rises of blood glucose (**e**) and its area under the curve (AUC, **f**) in ipGTT in C57BL/6J mice fasted 4 h. *n* = 7–8. **g** p.o. Allu failed to improve glucose tolerance in *Glp1r* KO mice fasted for 4 h (*n* = 6). **h**–**k** Allu potentiated insulin action to lower blood glucose (**h**) and its AUC (**j**) in ITT, and these effects were abolished in the presence of Ex(9-39) in C57BL/6J mice fasted 4 h (**i**, **j**) and in *Glp1r* KO mice (**k**). *n* = 5–11. **l**–**o** Allu suppressed blood glucose elevation (**l**) and its AUC (**n**) in PTT, and these effects were completely blocked in the presence of Ex(9-39) in C57BL/6J mice fasted overnight (**m**, **n**) and in *Glp1r* KO mice fasted overnight (**o**). *n* = 5–6. Different letters indicate *p* < 0.05 by two-way ANOVA followed by Tukey’s test (**e**), and *p < 0.05, **p < 0.01 by two-way ANOVA followed by Bonferroni’s test vs. saline group (**a**–**c**, **h**, **l**). In **d**, #*p* < 0.05 by repeated measures ANOVA followed by Dunnett’s test vs. 0 min in Allu group. In **f**, **j**, **n**, different letters indicate *p* < 0.05 by one-way ANOVA followed by Tukey’s test. Error bars are SEM

In i.p. glucose tolerance test (ipGTT) in C57BL/6J mice fasted overnight (16 h), p.o. d-allulose (1 g kg^−1^), administered 60 min before i.p. glucose (2 g kg^−1^) injection, did not influence basal blood glucose levels but markedly attenuated rises in blood glucose levels at 15, 30, and 60 min (Fig. 3a). Concomitantly, plasma insulin levels were slightly but significantly elevated at 15 min with d-allulose, compared with saline injection (Fig. 3b). In mice fasted 4 h, 1 g kg^−1^ d-allulose also markedly attenuated rises in blood glucose in ipGTT (Fig. 3c). Plasma insulin levels were not significantly different between d-allulose and saline groups at 0, 15, 30 min in ipGTT, however, in the presence of d-allulose insulin level significantly increased at 15 min compared to 0 min (Fig. 3d), suggesting an increased insulin secretory response to glucose.

To examine the involvement of GLP-1R signaling, Ex(9-39) (200 nmol kg^−1^) or saline was i.p. administered at −75 min, and d-allulose (1 g kg^−1^) or saline was administered at −60 min (Fig. 3e). The Ex(9-39) treatment, compared with saline, itself significantly elevated blood glucose levels (Fig. 3e) and area under the curve (AUC) of blood glucose levels during 0–120 min in ipGTT (Fig. 3f). Following treatment with Ex(9-39), the effects of d-allulose to lower blood glucose level and its AUC in ipGTT were blunted (Fig. 3e, f). Moreover, d-allulose (1 g kg^−1^) failed to alter blood glucose levels in *Glp1r* KO mice (Fig. 3g). These data indicated that oral d-allulose improved glucose tolerance via the GLP-1R.

The results that d-allulose moderately increased insulin release only at 15 min prompted us to explore possible additional mechanisms for the glycemic action of d-allulose. First, the effect of d-allulose on insulin action was investigated with an ITT. Oral d-allulose (1 g kg^−1^), injected 1 h prior to i.p. insulin (1 IU kg^−1^), enhanced the blood glucose lowering effect of insulin at 15–240 min, with statistically significant difference (vs. saline) at 120 min and later (Fig. 3h). The AUC of blood glucose levels during 0–240 min was significantly reduced in the presence of d-allulose (Fig. 3j). The potentiation of insulin action by d-allulose was blocked by treatment with 200 nmol kg^−1^ Ex(9-39) (Fig. 3i, j) and attenuated in *Glp1r* KO mice (Fig. 3k). The effect of d-allulose on glucose production was next investigated with a PTT, which indirectly assesses hepatic gluconeogenesis. Treatment with d-allulose (1 g kg^−1^) from −60 min robustly suppressed the rise in blood glucose level and its AUC during 0–120 min (Fig. 3l, n). The action of d-allulose to attenuate the blood glucose rises in PTT was counteracted by treatment with 200 nmol kg^−1^ Ex(9-39) (Fig. 3m, n) and absent in *Glp1r* KO mice (Fig. 3o). These results indicate that oral administration of d-allulose enhances insulin action and suppresses glucose production via the GLP-1R.

d-Allulose via i.p. route neither increased GLP-1 secretion (Supplementary Fig. 3a), nor influenced glucose tolerance, insulin action, or glucose production (Supplementary Fig. 3b–d). In ex vivo experiments, administration of d-allulose had no effect on glucose (11.2 mM)-induced insulin secretion from isolated pancreatic islets under static incubation (Supplementary Fig. 4), indicating lack of direct interaction of d-allulose with islets. These results demonstrate that d-allulose improves glucose tolerance via potentiation of GLP-1R signaling.

### d-Allulose reduces feeding via GLP-1R in HFD-fed mice

**Fig. 4:**
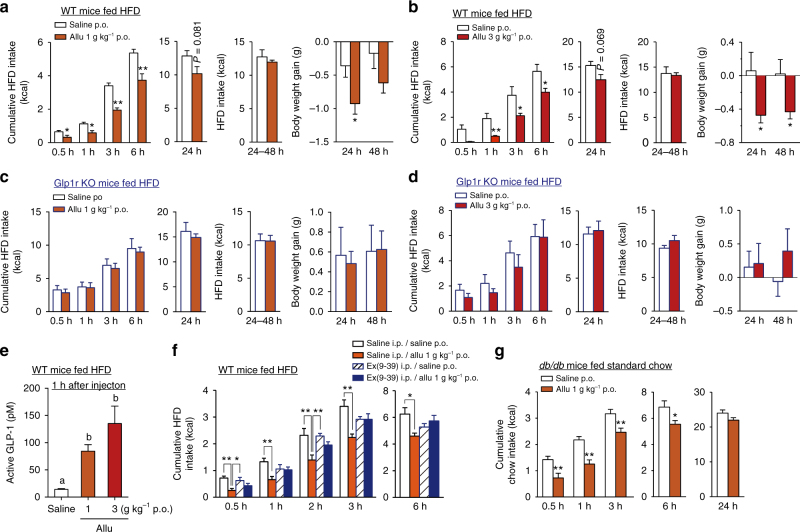
p.o. d-Allulose suppresses food intake in HFD-fed obese and diabetic *db/db* mice. **a**–**d** Cumulative HFD intake at 0.5, 1, 3, 6, and 24 h and for 24–48 h and body weight gain after p.o. administration of Allu (1 and 3 g kg^−1^) at 19:30 in HFD-fed obese C57BL/6J (wild-type, WT) fasted for 3 h (**a**, **b**) and HFD-fed *Glp1r* KO mice fasted for 3 h (**c**, **d**). *n* = 5–6. **e** Active GLP-1 level in portal vein 1 h after p.o. Allu in HFD-fed C57BL/6J mice fasted overnight. *n* = 5–6. **f** Treatment with 600 nmol kg^−1^ Ex(9-39) attenuated the effect of p.o. Allu to reduce HFD intake in HFD-fed obese mice. *n* = 6. **g** Cumulative standard chow intake after p.o. Allu injected at 10:00 in *db/db* mice fasted overnight. *n* = 9–10. In **a**, **b**, **g**, *p < 0.05 and **p < 0.01 by unpaired *t*-test in each time. In **e**, different letters indicate *p* < 0.05 and *p < 0.05, **p < 0.01 by one-way ANOVA followed by Tukey’s test. Error bars are SEM

We next examined the effects of d-allulose on food intake in obese and diabetic mice. In high-fat diet (HFD)-fed obese mice, p.o. administration of d-allulose at 1 g kg^−1^ (Fig. 4a) and 3 g kg^−1^ (Fig. 4b) at the dark period (DP) onset (19:30) significantly suppressed cumulative HFD intake at 0.5–6 h. Notably, cumulative food intake for 24 h tended to be suppressed without rebound for the following 24–48 h period (Fig. 4a, b), which was accompanied by reduction in body weight gain at 24 h and even 48 h (Fig. 4a, b). These acute anorexigenic and weight-reducing effects of d-allulose were not observed in *Glp1r* KO mice-fed HFD for 5 weeks or longer (Fig. 4c, d). HFD-fed obese mice responded to p.o. d-allulose with a rise in portal GLP-1 concentration to a level approximately twofold higher compared to standard chow-fed mice (Fig. 4e vs. Fig. 1h). Accordingly, a threefold higher dose (600 nmol kg^−1^) of Ex(9-39) was used in HFD-fed mice and it counteracted the anorexigenic effects of d-allulose (Fig. 4f). Furthermore, in *db/db* mice, a genetic obesity model due to a leptin receptor mutation, p.o. administration of d-allulose (1 g kg^−1^) suppressed food intake at 0.5–6 h in a similar manner to chow-fed and HFD-fed obese C57BL/6J mice (Fig. 4g vs. Figs. 1a and 4a). These data indicate that the actions of d-allulose to suppress food intake via GLP-1R signaling are preserved in multiple preclinical models of diabetes and obesity.

### Allu improves glucose tolerance via GLP-1R in HFD-fed mice

**Fig. 5:**
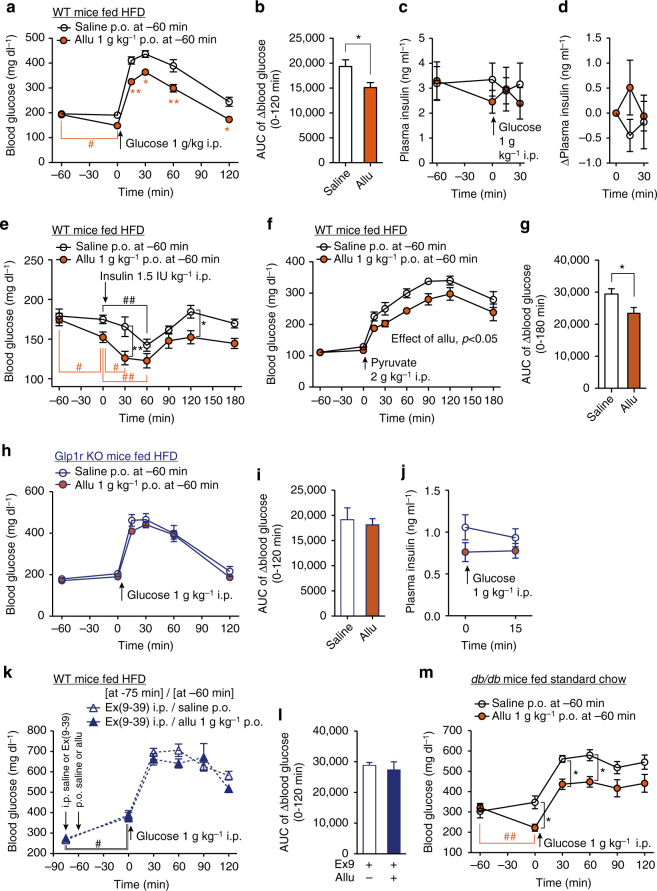
d-Allulose improves glucose tolerance via GLP-1R in obese and diabetic mice. Allu at 1 g kg^−1^ or saline was p.o. administered at 60 min prior to ipGTT (1 g kg^−1^, **a**–**d**, **h**–**m**), ITT (1.5 IU kg^−1^, **e**), and PTT (2 g kg^−1^, **f**, **g**) in HFD-fed obese C57BL/6J mice (WT), HFD-fed *Glp1r* KO mice or standard chow-fed diabetic *db/db* mice. **a**–**d** Blood glucose (**a**) and plasma insulin levels (**c**) after p.o. injection of Allu at −60 min and during ipGTT in HFD-fed obese and hyperglycemic C57BL/6J mice fasted for 4 h. **b** AUC for rise of blood glucose during 0–120 min. **d** Change of plasma insulin levels after ipGTT plotted from **c**. *n* = 6–10. **e** Blood glucose levels after p.o. Allu at −60 min and during ITT in HFD-fed mice fasted for 4 h. *n* = 6–7. **f**, **g** Blood glucose level and its AUC during 0–180 min in PTT in HFD-fed mice fasted overnight. *n* = 6–7. **h**–**j** In HFD-fed *Glp1r* KO mice fasted 4 h, blood glucose level (**h**) and its AUC (**i**) and plasma insulin levels (**j**) after p.o. administration of Allu and during ipGTT. *n* = 6. **k**, **i** Effect of 600 nmol kg^−1^ Ex(9-39) administered at −75 min on blood glucose level (**k**) and its AUC (**i**) in GTT in HFD-fed C57BL/6J mice fasted 4 h. **m** Blood glucose levels after p.o. Allu at −60 min and during ipGTT in *db/db* mice fasted for 4 h. *n* = 5. In **a**, **e**, **m**, *p < 0.05, **p < 0.01 by two-way ANOVA followed by Bonferroni’s test vs. saline group. In **a**, **e**, **k**, **m**, #*p* < 0.05, ##*p* < 0.01 by two-way ANOVA followed by Dunnett’s test vs. 0 min in each group. In **f**, effect of Allu treatment was significant with *p < *0.05 by two-way ANOVA. *p < 0.05 by unpaired *t*-test (**b**, **g**). Error bars are SEM

In HFD-fed mice with elevated basal glucose levels (~200 mg dl^−1^), p.o. administration of d-allulose (1 g kg^−1^) at −60 min significantly lowered basal blood glucose level at 0 min, and suppressed rises in blood glucose level at 15–120 min and its AUC for 0–120 min period in ipGTT (Fig. 5a, b). d-Allulose at −60 min tended to decrease plasma insulin at 0 min (Fig. 5c) and increase plasma insulin at 15 min after i.p. glucose injection (Fig. 5c, d), suggesting that d-allulose might partially restore the insulin secretory response to glucose.

In HFD-fed mice fasted moderately for 4 h, administration of saline at −60 min did not alter blood glucose at 0 min (Fig. 5e), and subsequent i.p. injection of insulin (1.5 IU kg^−1^) lowered blood glucose level at a rate slower than that in normal lean mice, without reaching significant difference at 30 min of ITT (Fig. 5e vs. Fig. 3h), suggesting insulin resistance. In contrast, administration of d-allulose at −60 min significantly attenuated basal glucose levels at 0 min (Fig. 5e). Moreover, in the presence of d-allulose, i.p. insulin (1.5 IU kg^−1^) significantly decreased blood glucose at 30 min (Fig. 5e). These results suggest that d-allulose significantly restored insulin action in HFD-fed mice. Moreover, single p.o. administration of d-allulose did not change blood glucose levels at 0 min in fasted (16 h) HFD-fed mice (Fig. 5f). Subsequently, the increases in blood glucose and AUC by challenge with pyruvate were significantly suppressed in the presence of d-allulose compared to saline (Fig. 5f, g). These data indicate that d-allulose significantly suppressed glucose production in HFD-fed mice with insulin resistance. Thus, d-allulose augmented insulin action, suppressed glucose production and enhanced insulin secretion in obese mice, complementary actions improving glucose tolerance.

In hyperglycemic *Glp1r* KO mice fed HFD for 5 weeks or longer, p.o. administration of d-allulose neither decreased the elevated basal blood glucose level, nor improved glucose tolerance in ipGTT (Fig. 5h, i). Insulin levels trended higher at 15 min of ipGTT after d-allulose in WT mice (Fig. 5d), but not in HFD-fed *Glp1r* KO mice (Fig. 5j). Furthermore, treatment with Ex(9-39) counteracted the effects of d-allulose to lower basal blood glucose (0 min) and to improve glucose tolerance under glucose challenge (0–120 min) in HFD-fed mice (Fig. 5k, l). These data indicate that d-allulose ameliorates basal hyperglycemia and impaired glucose tolerance (IGT) in HFD-fed mice via GLP-1R signaling.

The effect of d-allulose on glycemia was also examined in type 2 diabetic *db/db* mice with genetic inactivation of the leptin receptor, which exhibits obesity and insulin resistance. In *db/db* mice, p.o. administration of d-allulose (1 g kg^−1^) at −60 min significantly lowered elevated basal blood glucose level at 0 min and rises in blood glucose levels at 30 and 60 min during ipGTT (Fig. 5m). Blood glucose levels over the period till 60 min were reduced by d-allulose treatment.

### Chronotherapeutic effects of Allu on hyperphagic obesity

**Fig. 6:**
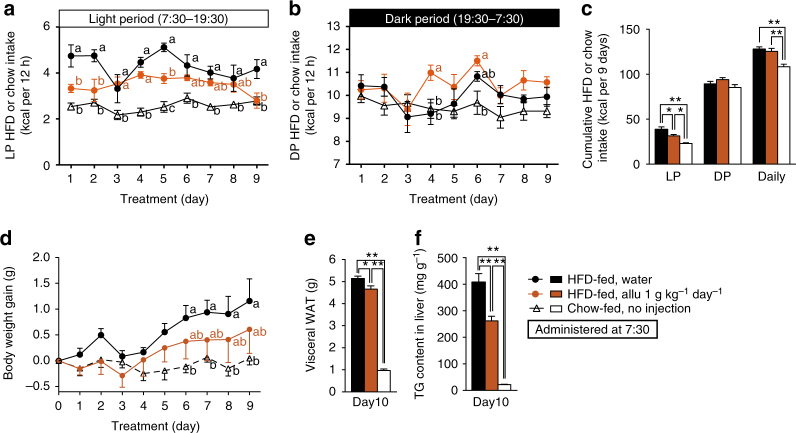
Chronotherapeutic effects of d-allulose (1 g kg^−1^ day^−1^) on hyperphagic obesity in HFD-fed mice. Subchronic treatment for 9 days of HFD-fed obese C57BL/6J mice with 1 g kg^−1^ day^−1^ p.o. Allu once daily at LP 7:30. **a**–**c** HFD-fed mice (HFD-fed), compared to C57BL/6J lean mice fed standard chow (Chow-fed), exhibited LP-selective hyperphagia accompanied by daily hyperphagia. Subchronic administration of Allu significantly suppressed LP (**a**, **c**), but not DP (**b**, **c**), and daily food intake (**c**), tended to attenuate body weight gain (**d**), and significantly decreased visceral WAT weight (**e**) and triacylglycerol content in liver (**f**). Visceral WAT weight was the sum of mesenteric, perirenal, and epididymal WAT. *n* = 5–6. In **a**, **b**, **d**, different letters *p < *0.05 by two-way ANOVA followed by Tukey’s test. In **c**, **e**, **f**, *p < 0.05, and **p < 0.01 by one-way ANOVA followed by Tukey’s test. Error bars are SEM

**Fig. 7:**
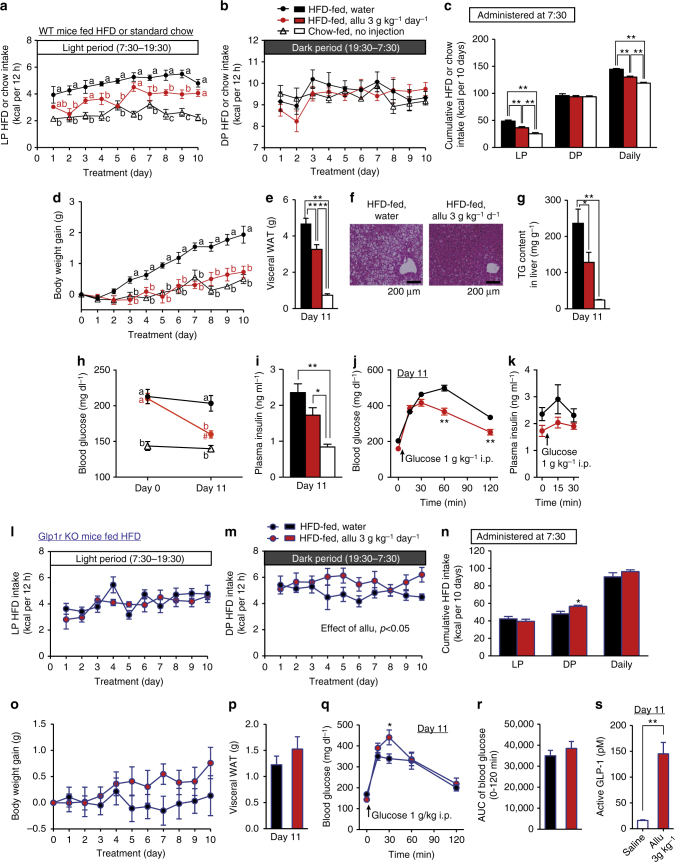
Chronotherapeutic effects of d-allulose (3 g kg^−1^ day^−1^) on hyperphagic obesity in HFD-fed mice. **a**–**k** HFD-fed obese C57BL/6J mice (HFD-fed) were subchronically treated for 10 days with p.o. Allu (3 g kg^−1^ day^−1^) or water once daily at LP 7:30. C57BL/6J lean mice fed standard chow (Chow-fed) were the lean control. Allu, compared to water, markedly attenuated increases in LP and daily HFD intake (**a**–**c**) and body weight gain (**d**). Subchronic Allu treatment ameliorated increased visceral WAT weight (**e**), hepatic steatosis (**f**), and elevated triacylglycerol content in liver (**g**) on Day 11. Scale bar, 200 µm. Allu treatment significantly ameliorated elevated basal blood glucose (**h**) with trend to attenuate hyperinsulinemia on Day 11 (**i**). In ipGTT at Day 11, rises in blood glucose at 60 and 120 min were markedly suppressed (**j**), and basal (0 min) and elevated plasma insulin levels at 15 and 30 min tended to decrease (**k**). **l**–**s** In HFD-fed *Glp1r* KO mice, the same subchronic Allu treatment did not alter LP HFD intake (**l**, **n**), slightly elevated DP HFD intake at Day 4 and later (**m**, **n**), and failed to significantly change body weight gain (**o**), visceral WAT weight on Day 11 (**p**), and blood glucose level (**q**) and its AUC (**r**) in ipGTT on Day 11 except an increase of blood glucose level at 30 min in ipGTT (**q**). Before sacrificing these *Glp1r* KO mice, portal vein was sampled 60 min after p.o. Allu (3 g kg^−1^) or saline and active GLP-1 was determined (**s**). *n* = 5–6. Different letters *p < *0.05 and **p < 0.01 by two-way ANOVA followed by Tukey’s test (**a**, **d**, **h**) or Bonferroni’s test (**j**, **q**). #*p < *0.01 by two-way ANOVA followed by Bonferroni’s test vs. Day 0 (**h**). In **m**, effect of Allu treatment was significant with *p < *0.05 by two-way ANOVA. *p < 0.05, and **p < 0.01 by one-way ANOVA followed by Tukey’s test (**c**, **e**, **g**, **i**). *p < 0.05, and **p < 0.01 by unpaired *t*-test vs. saline group (**n**, **s**). Error bars are SEM

Impaired diurnal feeding rhythm is implicated in development of obesity, diabetes, and metabolic syndrome in humans and rodents^3,4,28^. In rodents, the arrhythmic feeding is featured by hyperphagia during light period (LP), and its correction ameliorates obesity^3,4^. In the present study using obese mice fed HFD for 5 weeks or longer, food intake in LP (7:30–19:30) was approximately doubled, whereas that in dark period (DP, 19:30–7:30) unaltered, exhibiting LP-specific hyperphagia accompanied by daily hyperphagia (Figs. 6a–c and 7a–c). Hence, we examined whether subchronic administration of d-allulose daily at LP onset ameliorates LP hyperphagia, obesity, and IGT in HFD-fed mice.

Subchronic daily p.o. administration of d-allulose (1 and 3 g kg^−1^) at the LP onset 7:30 attenuated LP hyperphagia without altering DP food intake, thereby improving diurnal feeding rhythm in HFD-fed mice (Figs. 6a–c and 7a–c). With 3 g kg^−1^ d-allulose, this amelioration of LP hyperphagia was accompanied by suppression of daily hyperphagia (Fig. 7c). These changes in food intake were paralleled by amelioration of elevated body weight gain (Figs. 6d and 7d), visceral fat weight (Figs. 6e and 7e and Supplementary Fig. 5a–c), and liver triacylglycerol (TG) content (Figs. 6f and 7g) and hepatic steatosis (Fig. 7f) without altering weights of several peripheral organs (Supplementary Fig. 5d–i) in HFD-fed mice. Administration of 1 g kg^−1^ d-allulose for 9–10 days ameliorated arrhythmic feeding and visceral obesity (Fig. 6 and Supplementary Fig. 6a–e), and tended to increase energy expenditure, fat oxidation and uncoupling protein-1 (UCP-1) expression without altering locomotor activity (Supplementary Fig. 6f–j). These data suggest that d-allulose may moderately enhance energy expenditure, which might additionally contribute to attenuation of visceral obesity, in agreement with previous reports that d-allulose increases energy expenditure^29^. d-Allulose treatment at 3 g kg^−1^ day^−1^ for 10 days significantly reduced the elevated basal blood glucose levels and tended to attenuate hyperinsulinemia in HFD-fed mice fasted 4 h (Fig. 7h, i). In ipGTT performed 1 day after termination of d-allulose treatment for 10 days, rises in blood glucose at 60 and 120 min were markedly suppressed (Fig. 7j), and both basal (0 min) and elevated plasma insulin levels (15 and 30 min) tended to be lower (Fig. 7k). The reductions in both blood glucose and plasma insulin levels suggested that subchronic d-allulose treatment improved glucose tolerance at least partly by ameliorating insulin resistance in HFD-fed mice, consistent with previous findings^17^.

**Fig. 8:**
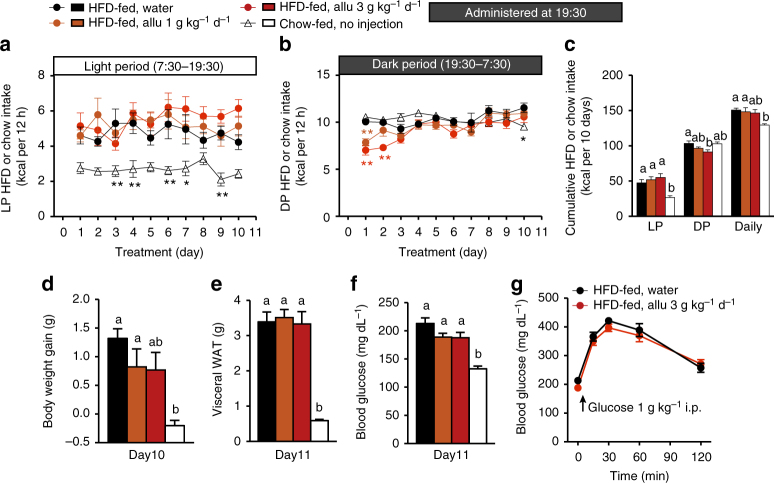
DP d-allulose administration fails to ameliorate LP-hyperphagia, obesity and glucose intolerance. Effects of subchronic p.o. administration of Allu (1 or 3 g kg^−1^) once daily at DP onset (19:30) for 10 days in HFD-fed obese mice (HFD-fed). Lean C57BL/6J fed standard chow (Chow-fed) without administration was the control for hyperphagic obesity. Time course of LP (**a**) and DP HFD intake (**b**), and cumulative HFD intake for 10 days (**c**). Body weight gain (**d**), visceral white adipose tissue (WAT) (**e**), and basal blood glucose level (**f**) in mice fasted 4 h at Day 10. Visceral WAT included mesenteric, perirenal and epididymal fat (**g**). *n* = 6–7. The blood glucose following i.p. injection of glucose in HFD-fed mice treated with water and 3 g kg^−1^ day^−1^ Allu (**g**). *p < 0.05 and **p < 0.01 by two-way ANOVA followed by Tukey’s test vs. HFD-fed, water group (**a**, **b**). Different letters indicate *p* < 0.05 by one-way ANOVA followed by Tukey’s test (**c**–**f**). Error bars are SEM

*Glp1r* KO mice fed HFD exhibited LP hyperphagia (3.34 ± 0.25 kcal with HFD vs. 1.89 ± 0.33 kcal with chow, *n* = 12, *p* < 0.05 by unpaired *t*-test). In HFD-fed *Glp1r* KO mice, subchronic (10 days) p.o. d-allulose (3 g kg^−1^) administration once daily at 7:30 failed to suppress LP hyperphagia and daily food intake, while it slightly elevated DP food intake (Fig. 7l–n). The d-allulose treatment did not significantly change body weight gain, visceral white adipose tissue (WAT) weight, blood glucose levels and AUC in ipGTT (Fig. 7o–r) except that at 30 min (Fig. 7q) in HFD-fed *Glp1r* KO mice, while it markedly increases GLP-1 secretion, as detected by portal GLP-1 levels (Fig. 7s). Hence, subchronic d-allulose treatment at early LP ameliorates LP-specific hyperphagia, obesity, adiposity and IGT in HFD-fed mice via GLP-1R signaling. Importantly, when d-allulose was subchronically administered at DP onset (19:30), it failed to ameliorate LP and daily hyperphagia, obesity, adiposity and IGT (Fig. 8). These data indicate that timing of d-allulose administration is critical for its action to ameliorate LP-hyperphagia and metabolic disorders in HFD-fed obese/IGT mice.

### Feeding and glycemic effects of Allu require vagal afferents

**Fig. 9:**
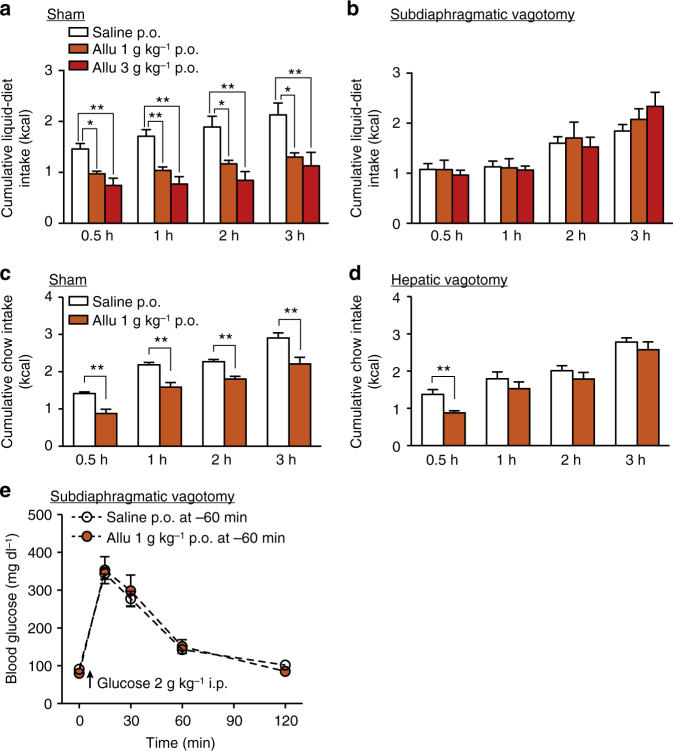
Subdiaphragmatic and hepatic vagotomy counteract metabolic actions of d-allulose. **a**, **b** Allu (1 and 3 g kg^−1^, p.o.) reduced food intake in sham-operated (**a**, *n* = 6–7) but not subdiaphragmatic vagotomized C57BL/6J mice (**b**, *n* = 6–12) fasted overnight (16 h). *p < 0.05 and **p < 0.01 by one-way ANOVA followed by Tukey’s test. **c**, **d** Anorexigenic effect of Allu (1 g kg^−1^, p.o.) in sham-operated mice fasted overnight (**c**, *n* = 10–11) was blunted in hepatic vagotomized mice (**d**, *n* = 6) for 1–3 h, but not 0.5 h, after injection. *p < 0.05 and **p < 0.01 by unpaired *t*-test. **e** Allu (1 g kg^−1^, p.o.) failed to improve glucose tolerance in subdiaphragmatic vagotomized mice fasted for 4 h (*n* = 6). Error bars are SEM

The effects of d-allulose shown here may involve the brain, however, d-allulose does not pass through BBB^30^, raising a question whether d-allulose exerts its effects at least partly through vagal afferent nerves^31,32^. The effects of 1 and 3 g kg^−1^ d-allulose to suppress food intake for 0.5–3 h were abolished in mice with subdiaphragmatic vagotomy (Fig. 9a vs. Fig. 9b). When the hepatic branch of vagal afferents, which sense gastrointestinal hormones^25^, was selectively denervated, the anorexigenic effect of 1 g kg^−1^ d-allulose for 1–3 h was blocked, whereas that at 0.5 h was unaltered (Fig. 9c vs. Fig. 9d). Subdiaphragmatic vagotomy also attenuated the ability of d-allulose to enhance glucose tolerance (Fig. 9e vs. Fig. 3a, c, e). These results indicated that the effects of d-allulose on food intake and glucose metabolism are largely mediated by vagal afferent nerves.

### d-Allulose activates vagal afferent neural pathways

**Fig. 10:**
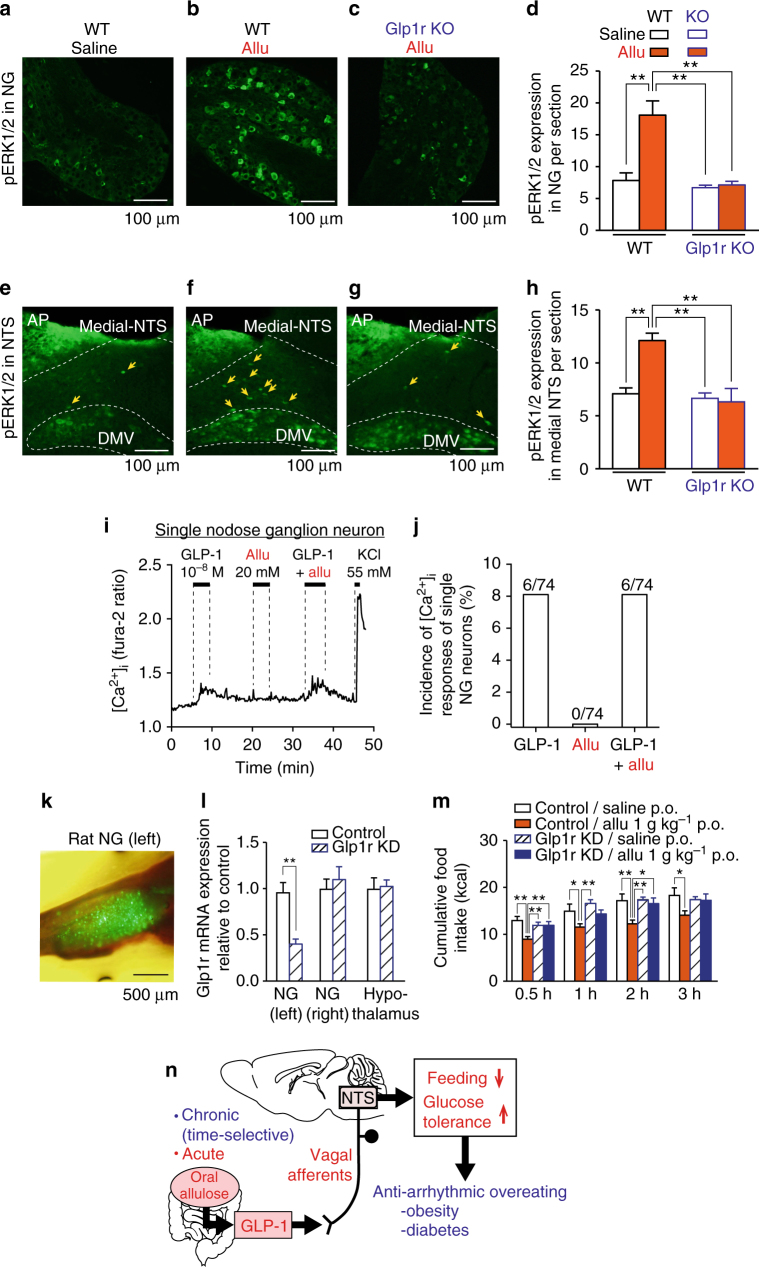
p.o. d-Allulose activates vagal afferents and NTS via GLP-1R. **a**–**h** Allu (1 g kg^−1^, p.o.), compared to saline, induced ERK1/2 phosphorylation in vagal afferent nodose ganglion (NG) (**a**–**d**) and medial NTS (**e**–**h**) in WT mice (*n* = 5) but not *Glp1r* KO mice (*n* = 6). Scale bar, 100 µm. **i**, **j** GLP-1 at 10^−8^ M increased [Ca^2+^]_*i*_ in 6 of 74 (8.1%) single neurons isolated from nodose ganglion. Allu at 20 mM neither induced [Ca^2+^]_*i*_ nor potentiated GLP-1-induced [Ca^2+^]_*i*_ increases in nodose ganglion neurons. The trace in **i** was representative of six neurons. **k**–**m** Vagal afferent-specific *Glp1r* knockdown (KD) attenuated the anorexigenic effect of Allu (1 g kg^−1^, p.o.) in rats. Visualization of ZsGreen1 expression in the whole left NG of a rat injected with AAV9-*Glp1r*-shRNA particles (**k**). Relative expression of GLP-1 mRNA in the left NG (AAV injected), right NG (not injected) and hypothalamus in control and *Glp1r* KD rats (**l**). The cumulative food intake after Allu (1 g kg^−1^, p.o.) or saline administration in control and *Glp1r* KD rats fasted 16 h (**m**). *n* = 11. *p < 0.05 and **p < 0.01 by one-way ANOVA followed by Tukey’s test. Scale bar, 500 µm. **n** Proposed mechanism for the action of oral Allu. Acute Allu suppresses feeding and improves glucose tolerance via GLP-1-mediated vagal afferent pathway. When time-selectively administered, chronic d-allulose corrects arrhythmic overeating, obesity and diabetes, suggesting chronotherapeutic potential to enhance GLP-1R signaling to treat metabolic disorders. Error bars are SEM

We next examined whether p.o. d-allulose activates vagal afferent neurons and nucleus tractus solitarius (NTS) to which vagal afferents project and, if so, whether these effects are mediated via GLP-1R signaling. Immunohistochemical staining for cellular/neuronal activation markers, phosphorylation of extracellular signal-regulated kinase 1 and 2 (pERK1/2) and c-Fos^27,33^, were examined. P.o. administration of d-allulose (1 g kg^−1^) induced expression of pERK1/2 in vagal afferent nodose ganglion neurons and in NTS of WT C57BL/6J mice, but not in *Glp1r* KO mice (Fig. 10a–h). Similarly, p.o. d-allulose (1 g kg^−1^) increased c-Fos expression in NTS (Supplementary Fig. 7). These results indicated that p.o. d-allulose GLP-1R dependently activates vagal afferent neurons and NTS, the areas implicated in regulation of feeding^27,34^.

### GLP-1 but not Allu directly acts on vagal afferent neurons

We examined whether d-allulose activates nodose ganglion (NG) neurons that comprise vagal afferents. d-Allulose (20 mM) failed to directly increase cytosolic Ca^2+^ concentration ([Ca^2+^]_*i*_) in single neurons isolated from the NG of vagal afferents (Fig. 10l, j). By contrast, GLP-1 (10^−8^ M) increased [Ca^2+^]_i_ in 8.1% of single NG neurons (Fig. 10l, j). d-Allulose had no effect on the GLP-1-induced [Ca^2+^]_i_ increase (Fig. 10l, j). Thus, d-allulose activates GLP-1 secretion, which in turn directly interacts with vagal afferent neurons to induce [Ca^2+^]_*i*_ signaling.

### NG-specific Glp1r knockdown blunts anorectic action of Allu

The anorexigenic effect of d-allulose was attenuated by vagotomy of hepatic branch (Fig. 9c, d), which suggested that GLP-1 might act through the hepatic vagal branch. We examined the effect of d-allulose on food intake in rats whose *Glp1r* was knocked down selectively in the ventral trunk of vagal afferents including hepatic branch, which was achieved by microinjection of AAV9-*Glp1r*-shRNA vector into the left NG. Three weeks after the microinjection, successful infection was confirmed by visualizing ZsGreen1 expression in whole left NG (Fig. 10k), but not in right intact NG. GLP-1R mRNA expression in the left NG significantly decreased in *Glp1r* knockdown (KD) rats to 41% of the level in control rats injected with AAV9-Scrambled (Scr)-shRNA vector (Fig. 10l). Neither GLP-1R mRNA expression in the right NG and hypothalamus nor anorexigenic CCK-A receptor mRNA expression in the left and right NGs, was altered in *Glp1r* KD rats (Fig. 10l and Supplementary Fig. 8). These results indicated that the left NG-specific KD of GLP-1R was achieved by the *Glp1r*-shRNA construct used.

In these *Glp1r* KD rats, p.o. d-allulose at 1 g kg^−1^ failed to significantly suppress food intake at 0.5, 1, 2, and 3 h after injection (Fig. 10m), while it significantly reduced food intake at all time points in control AAV9-Scr-shRNA-injected rats. These results collectively demonstrate that GLP-1R signaling via vagal afferents is necessary for the anorexigenic effect of oral d-allulose.

## Discussion

In the present study, we found that single p.o. administration of d-allulose stimulated GLP-1 release, inhibited food intake, and promoted glucose tolerance in chow-fed and HFD-fed obese/hyperglycemic mice. d-Allulose promoted glucose tolerance by enhancing insulin release and action and inhibiting glucose production. In HFD-fed obese mice, subchronic p.o. administration of d-allulose once daily at LP onset (7:30) attenuated LP hyperphagia to restore diurnal feeding rhythm, and thereby suppressed daily hyperphagia, obesity, adiposity, and IGT. Conversely, subchronic d-allulose at DP onset (19:30) was ineffective in attenuating LP hyperphagia and exerting the metabolic effects, showing chronotherapeutic action of d-allulose. These effects of d-allulose to attenuate feeding and promote glucose tolerance were diminished by a GLP-1R antagonist, and attenuated in *Glp1r* KO mice, and by vagotomy. Furthermore, the *Glp1r* KD specifically in the ventral trunk of vagal afferents eliminated the anorexigenic effect of d-allulose. These results identified oral d-allulose as a GLP-1 releaser that attenuates feeding and promotes glucose tolerance in lean and hyperglycemic/obese mice via a vagal afferent-mediated pathway. Furthermore, when time-specifically administered, chronic d-allulose corrected arrhythmic overeating, obesity and diabetes, suggesting a potential for chronotherapy (Fig. 10n).

The d-allulose-induced suppression of food intake at 0.5 h was not counteracted by i.p. pretreatment with Ex(9-39) (Fig. 2a, b), whereas it was blunted in *Glp1r* KO mice (Fig. 2f, g), vagal afferent-specific *Glp1r* KD rats (Fig. 10m), and vagotomized mice (Fig. 9b). These results suggest that Ex(9-39) at the dose used is unable to antagonize GLP-1R at an early period of 0.5 h possibly due to the following reasons. Ex(9-39) may not be fully absorbed at this early time point. Alternatively, i.p. administration may result in a substantial rise of Ex(9-39) in the general circulation but not in the microcirculation in the specific tissue where GLP-1 is sensed, including the intestine and portal.

Surprisingly, d-allulose stimulated release of GLP-1, but not PYY. We speculate that d-allulose may activate a subset of L-cells in the proximal or middle small intestine where the majority of L-cells express GLP-1, while most PYY-expressing L-cells appear in the distal ileum and large intestine^35,36^. Alternatively, d-allulose might evoke cellular signaling selectively linked to release of GLP-1, but not PYY, from L-cells expressing one or both peptides. The mechanism underlying selective release of GLP-1 by d-allulose remains to be elucidated.

The absence of glucose-induced GLP-1 secretion could be due to that the dose of glucose used (1 or 3 g kg^−1^) is lower than that (higher than 5 g kg^−1^) previously reported to increase plasma GLP-1^37–40^. Although several studies documented GLP-1 secretion with glucose at 2–4 g kg^−1^ ^41,42^, others failed to observe the increment of GLP-1 with oral glucose at 2 or 3 g kg^−1^ ^40,43^. Alternatively, the time point for collecting the blood sample (60 min after oral load) might be too late to detect increase in plasma GLP-1, which was observed 5–10 min after oral glucose load in other reports^38,40,44,45^. Therefore, glucose might transiently activate proximal L cells, and is subsequently absorbed immediately in the proximal small intestine. Because d-allulose is slowly absorbed in the intestine compared to glucose^30^, it would stay in the intestinal lumen longer to stimulate L-cells.

The LP hyperphagia causes obesity in rodents^3,4^. In our study, d-allulose, administered at LP onset but not DP onset, ameliorated LP hyperphagia and visceral obesity in HFD-fed obese mice. Detailed mechanisms for the time-dependent effects of d-allulose remain unknown. However, since d-allulose inhibits food intake significantly for 0.5–6 h but not 24 h (Figs. 1a and 4a, b) and elevates plasma GLP-1 level for 0.5–3 h (Fig. 1d), d-allulose administered at early LP may be able to effectively suppress LP hyperphagia. Alternatively, the feeding center machinery whose impairment causes LP hyperphagia may have sensitivity to and be corrected by d-allulose administered in early LP. Notably, inversely to the correction of LP hyperphagia by d-allulose-GLP-1, *Glp1r* KO mice displayed deterioration of LP hyperphagia (Fig. 7n). These results collectively suggest that the physiological GLP-1 released postprandially serves to produce feeding rhythm, and that pharmacologic d-allulose and GLP-1, when administered in the time period with impaired GLP-1 rise, can ameliorate feeding arrhythmia.

The present results support that the release of GLP-1 by oral d-allulose activates vagal afferents, which in turn suppresses food intake and improves glucose metabolism (Fig. 10n). This is in consistent with previous reports that neural pathways contribute to the effect of GLP-1 and DPP-4 inhibitors on food intake and glucose metabolism^22,23,25,46^. This vagal afferent-mediated link of endogenous GLP-1 to food intake and glucose metabolism is consistent with several previous reports. Vagal afferents-specific *Glp1r* knockdown resulted in elevation of food intake for 1 h after overnight fasting and reduction of meal-evoked insulin secretion^23^. A study using β cell-specific *Glp1r* knockout showed that oral glucose- or DPP-4 inhibitor-induced elevation of endogenous GLP-1 improves glucose metabolism via extra-islet GLP-1Rs^47^. Intraduodenal infusion of metformin lowered hepatic glucose production via GLP-1R signaling and neuronal pathway including vagal afferents^21^.

We have demonstrated that oral d-allulose ameliorates obesity and diabetes via GLP-1 release relayed to vagal afferent signaling. Currently, two incretin-based medicines, DPP-4 inhibitors and GLP-1R agonists, are clinically available. DPP-4 inhibitors expand the life-span of physiological GLP-1 and GIP, and thereby lower blood glucose. However, they are incapable of reducing body weight, possibly due to elevation of GIP that promotes adiposity^48–50^. GLP-1R agonists ameliorate both diabetes and obesity, decrease cardiovascular events, stroke and nephropathy in clinical trials^8,9^ and ameliorate dementia and stroke in preclinical studies^51^. However, GLP-1R agonists also elicit adverse effects including a rise in heart rate, nausea and vomiting^6,10,11^, possibly by passing through the BBB and directly acting on neurons in the brain^13,52^. In contrast, the present study showed that d-allulose, a substance incapable of penetrating BBB^30^, ameliorated hyperphagia and obesity without inducing aversive behavior. This property may be due to the action of d-allulose to stimulate release of intestinal GLP-1, which interacts with restricted targets including the hepatic and/or intestinal branches of vagal afferents that serve to inform specific brain regions and peripheral organs to ameliorate hyperphagia, obesity and hyperglycemia^21,23^. This notion fits with a previous report that endogenous GLP-1 is sensed largely by the vagal afferents innervating the portal/liver areas^25^ and gastrointestinal tract^21^. Together with the fact that no appreciable side effects have been reported for d-allulose^18^, d-allulose may provide a selective and safe treatment and/or prevention for obesity and diabetes.

We found that administration of d-allulose significantly lowered basal glucose level in hyperglycemic (>200 mg dl^−1^) HFD-fed mice and *db/db* mice, while it did not alter basal glucose level (around 100 mg dl^−1^) in normal glycemic chow-fed mice. The results indicate the ability of d-allulose to ameliorate hyperglycemia without risk of hypoglycemic events, suggesting a therapeutic advantage. This specific effect could be produced by the acute d-allulose action to ameliorate insulin resistance in HFD-fed hyperglycemic mice (Fig. 5e). Moreover, this glucose-dependent property of d-allulose may be due to its principal action to stimulate release of GLP-1 that works glucose dependently.

Since d-allulose has sweet taste and an anorexigenic effect, consumption of d-allulose can decrease calorie intake while maintaining the pleasure of eating. The safety of d-allulose as dietary supplements has been approved by the US Food and Drug Administration (FDA, GRAS Notice No. GRN 498). Therefore, oral d-allulose may provide an efficacious, readily-applicable, and safe therapy to ameliorate obesity, diabetes, metabolic syndrome, and possibly associated cardiovascular/cerebral disorders.

## Methods

### Animal

Male C57BL/6J mice (H2b) and male Wistar rats were obtained from Japan SLC (Shizuoka, Japan) and male *db/db* mice were from CLEA Japan (Tokyo, Japan). The *Glp1r*^−*/*−^ C57BL/6J (*Glp1r* KO) mice generated and provided by Dr. D.J. Drucker at University of Toronto and Dr. Y. Yamada at Akita University^26^. The animals were housed in individual cages for at least 1 week under controlled temperature (23 ± 1 °C), humidity (55 ± 5%), and lighting (light on at 7:30 and off at 19:30). Standard chow (CE-2, CLEA japan) and water were available ad libitum. Mice and rats (8- to 16-weeks-old) were sufficiently habituated to handling before experiments.

Male C57BL/6J mice (35–45 g, 15- to 23-weeks-old) fed HFD, HFD-32 (CLEA Japan, 57% of total calorie from fat), for 40–100 days were used as the mouse model of diet-induced obesity. The HFD feeding for 30 days made the mice overweight and hyperglycemic compared with control mice fed standard chow (chow; CE-7, CLEA Japan) (BW; 32.5 ± 0.60 g vs. 24.5 ± 0.19 g, blood glucose; 190 ± 6.3 mg dl^−1^ vs. 120 ± 4.0 mg dl^−1^ in HFD- and chow-fed mice, respectively). The *Glp1r* KO male mice (24–28 g, 18- to 25-weeks-old) fed HFD for 35–65 days, exhibited lesser degrees of obesity^53^ but similar hyperglycemia.

Animal experiments were carried out under approval by the Institutional Animal Experiment Committee of the Jichi Medical University, and in accordance with the Institutional Regulation for Animal Experiments and Fundamental Guideline for Proper Conduct of Animal Experiment and Related Activities in Academic Research Institutions under the jurisdiction of the Ministry of Education, Culture, Sports, Science and Technology.

### Chemicals

d-Allulose was provided by Kagawa University Rare Sugar Research Center and Matsutani Chemical Industry Co. Ltd. The purity of d-allulose was higher than 98%. d-glucose, d-mannitol, and lithium chloride were purchased from Wako Pure Chemical Industries, Ltd. (Osaka, Japan). GLP-1(7-36 amide) and oxytocin were from Peptide Institute (Osaka. Japan). Porcine insulin was from Sigma (MO), and exendin(9-39) (Ex(9-39)) was from Abgent (CA).

### Measurement of food and water intake

We performed feeding experiments with two protocols. In first protocol, the male mice were deprived of food from 18:00 (16 h fasting) with free access to water 1 day before the experiment. On next day at 9:50, d-allulose (0.3–3 g kg^−1^, 10 ml kg^−1^), d-glucose (1 g kg^−1^) or saline (10 ml kg^−1^) was peroral (p.o.) or i.p. administrated, and at 10:00 standard chow (chow; CE-2) or HFD (HFD-32) was given. In second protocol, feeding experiments were started at 19:30 in male mice which had been deprived of food for 3 h (16:30–19:30) with free access to water. Then cumulative food and water intake for the following 0.5, 1, 2, 3, 6, and 24 h was measured by subtracting uneaten food/water from initially premeasured food/water after administration and checking the food spillage. In C57BL/6J mice receiving subdiaphragmatic vagotomy and sham operation, liquid diet (Chilmil, Morinaga, Tokyo, Japan) was given. Food intake data were expressed as energy intake based on the following conversion: CE-2 3.45 kcal g^−1^, HFD-32 5.08 kcal g^−1^, and liquid diet 0.644 kcal g^−1^. Ex(9-39) (200 or 600 nmol kg^−1^, 5 ml kg^−1^) or saline (5 ml kg^−1^) was i.p. administered 15 min before oral injection of d-allulose.

### Conditioned taste aversion test

Conditioned taste aversion test was performed as previously reported^27^. To accustom mice to water deprivation schedule, male C57BL/6J lean mice were allowed to access to two water bottles for 2 h (10:00–12:00) for 5 days. On the 6th day, mice were given 0.15% saccharine instead of water for 0.5 h, and then injected with saline (10 ml kg^−1^, p.o.), d-allulose (1 and 3 g kg^−1^, 10 ml kg^−1^, p.o.), or lithium chloride (LiCl, 3 nmol, 20 ml kg^−1^, i.p.). The 7th day was the rest day when mice had free access to normal water for 2 h. On the 8th day, two-bottle preference (0.15% saccharine vs. water) test was performed for 0.5 h. Conditioned taste aversion was determined as saccharine preference ratio, saccharine intake/total intake.

### Measurements of urine volume, osmolality, and excretion

Male C57BL/6J lean mice were housed in individual metabolic cages for 5 days to adapt to the environment. Subsequently, d-allulose (1 or 3 g kg^−1^) or saline was administered p.o., urine was collected and volume measured over 24 h. Urine osmolality was determined by Fiske Micro-Osmometer (Advanced instruments, MA). Urinary glucose and creatinine were measured by colorimetric-enzymatic method using Wako Autokit glucose C2 (Wako) and L-type Wako CRE (Wako), respectively. Urinary sodium, potassium, and chloride were quantified by DRI-CHEM 800 (Fujifilm, Tokyo, Japan).

### Gastrointestinal hormones assay in portal vein plasma

The blood samples were collected from the portal vein of male mice fasted overnight (18:00 to next 10:00) under isoflurane anesthesia at 0, 1, 2, and 3 h after p.o. administration of d-allulose (0.3 and 1 g kg^−1^, 10 ml kg^−1^) or saline (10 ml kg^−1^), respectively. The sampling syringe contained heparin (final concentration; 50 IU ml^−1^), aprotinin (final concentration; 500 kIU ml^−1^), and DPP-IV inhibitor vildagliptin (final concentration; 10 µM). Plasma was collected after centrifugation (3000 × *g*, 10 min at 4 °C) and stored at –80 °C until assay. Active GLP-1, total GIP, PYY, and CCK levels were measured using GLP-1 (Active) ELISA (EGLP-35K; Millipore), Rat/Mouse GIP (total) ELISA (EZRMGIP-55K; Millipore), Mouse/Rat PYY EIA (YK081, Yanaihara Institute, Inc.), and Human/Rat/Mouse Cholecystokinin Octapeptide (26-33, non-sulfated) EIA kits (Phoenix Pharmaceuticals, Inc.), respectively.

### Glucose, insulin, and PTTs

Male C57BL/6J, *Glp1r* KO, and *db/db* mice were fasted overnight (18:00 to next 10:00) or for 4 h (9:00–13:00) in GTT, for 4 h in ITT, and overnight in PTT. Basal glucose levels in blood samples obtained from tail vein were determined by GlucoCard DIA meter (Arkray, Tokyo, Japan). Then, d-allulose (1 g kg^−1^, 10 ml kg^−1^) or saline (10 ml kg^−1^) was administered orally or intraperitoneally at 60 min prior to each test. Blood was collected from tail vein at 0 min, followed by i.p. injection of d-glucose (1 or 2 g kg^−1^, 10 ml kg^−1^), insulin (1.0 or 1.5 IU kg^−1^, 10 ml kg^−1^) or sodium pyruvate (2 g kg^−1^, 10 ml kg^−1^), and blood samples were collected at 15–240 min. Blood samples were collected from tail vein using heparinized capillary glass, and plasma glucose and insulin were determined by Wako Autokit glucose C2 (Wako) and insulin ELISA kit (Morinaga, Yokohama, Japan). Ex(9-39) (200 or 600 nmol kg^−1^, 5 ml kg^−1^) or saline (5 ml kg^−1^) was i.p. administered 15 min before d-allulose injection.

### Measurement of insulin secretion from isolated islets

Measurement of insulin release from isolated islets under static incubation was performed following previous reports^54^. Male C57BL/6J mice were anaesthetized with i.p. pentobarbital (80 mg kg^−1^), followed by injection of collagenase (1.14 mg ml^−1^; Sigma-Aldrich) into the common bile duct. Pancreas was dissected out and incubated at 37 °C for 16 min. Islets were hand collected under a microscope. Groups of ten size-matched islets were incubated at 37 °C in HEPES-buffered Krebs-Ringer bicarbonate buffer (HKRB) composed of (in mM) 4.7 KCl, 1.2 KH_2_PO_4_, 129 NaCl, 5 NaHCO_3_, 1.2 MgSO_4_, 1.8 CaCl_2_, and 10 HEPES, with pH adjusted at 7.4 using NaOH supplemented with 2.8 mM glucose, followed by test incubation for 1 h with 2.8 or 11.2 mM glucose in the absence or presence of d-allulose (20 mM) or d-mannitol (20 mM). Insulin levels were determined by ELISA (Morinaga).

### Subchronic administration of d-allulose to HFD-fed mice

Male C57BL/6J wild-type mice and male *Glp1r* KO mice fed HFD for 5 weeks or longer were orally treated with d-allulose (1 or 3 g kg^−1^) or water (10 ml kg^−1^) once daily at the onset of LP (7:30) or DP (19:30) for 9 or 10 days. Male lean C57BL/6J mice fed standard chow (chow; CE-7, CLEA Japan) without oral injection were used as the control. Body weight and food intake at LP onset and DP onset were measured every day.

On Day 0 and Day 11 of the treatment with 3 g kg^−1^ d-allulose, glucose levels in the tail vein blood were measured with GlucoCard DIA meter in mice fasted 4 h (9:00–13:00). In GTT performed on Day 11, HFD-fed mice treated with d-allulose or water were i.p. injected with glucose (1 g kg^−1^). Glucose and insulin levels in the tail vein blood were determined by GlucoCard DIA meter and insulin ELISA kit (Morinaga).

On Day 11, peripheral organs (heart, spleen, kidney, pancreas, liver, mesenteric, perirenal and epididymal white adipose tissue, interscapular brown adipose tissue) were collected and their wet organ weights measured.

A liver sample was fixed with 4% paraformaldehyde overnight at 4 °C and embedded into paraffin. The liver sections were stained with Mayer’s hematoxylin and eosin solution. To measure triacylglycerol (TG), dissected liver cubes were extracted using chloroform/methanol (2:1) for 48 h, organic solvents were evaporated under N_2_ stream, and the crude lipids were re-suspended in isopropanol. TG concentrations in the solution were measured by TG specific enzymatic kit (Wako, Osaka, Japan). TG contents were normalized to the weight of liver.

Interscapular brown adipose tissue was homogenized in Tris-ethylenediaminetetraacetic acid (EDTA) buffer (10 mM Tris and 1 mM EDTA, pH 7.4) with 1% protease inhibitor (Nacalai Tesque, Kyoto, Japan) on ice. After centrifugation at 800 *× g* for 10 min at 4 °C, the supernatant was obtained. The total protein content was determined with BCA protein assay (TaKaRa, Shiga, Japan). Protein (5 µg) was separated using 12% SDS-PAGE and transferred to polyvinylidene fluoride membrane (Immobilon; Millipore, Tokyo, Japan). The membranes were subsequently probed with antibodies for UCP1 (662045, Millipore, 1:2000). A β-actin antibody (A5441, Sigma, 1:4000) was used as a loading control. Proteins were detected using horseradish peroxidase (HRP)-conjugated IgG secondary antibody (#7074, Cell Signaling and 62-6520, Thermo Fisher Scientific) and SignalFire ECL Reagent (Cell Signaling). Uncropped full-length images of immunoblots are shown in Supplementary Fig. 9.

### Measurements of energy expenditure and locomotor activity

HFD-induced obese C57BL/6J male mice were placed in individual small acryl calorimeter chambers with free access to HFD and water, and respiratory gas (O_2_ and CO_2_) were measured every 5 min for each individual mouse by indirect calorimetry system connected to mass spectrometer (Arco2000, ArcoSystem, Chiba, Japan). After 3 days of adaptation, d-allulose (1 g kg^−1^) or water (10 ml kg^−1^) was orally administered once daily at LP onset (7:30) for 10 days, and oxygen consumption (VO_2_) and carbon dioxide production (VCO_2_) were synchronously measured for 10 days. Respiratory exchange ratio (RER) was determined by the ratio VCO_2_/VO_2_. Energy expenditure (EE) was calculated as EE = (3.85 + 1.232 × RER) × VO_2_. Total carbohydrate consumption (CHO) and fat consumption (FAT) were calculated using the stoichiometric equations of Frayn as follows: CHO = 4.51 × VCO_2_−3.18 × VO_2_ [mg min^−1^], and FAT = 1.67 × (VO_2_−VCO_2_) [mg min^−1^]. Locomotor activity was estimated by the number of infrared beams broken in both *X* and *Y* directions using activity monitoring system (ACTIMO-100; Shinfactory, Fukuoka, Japan) combined with individual calorimeter chambers.

### Immunohistochemistry for pERK1/2 and c-Fos

Extracellular signal-regulated kinase phosphorylation (pEKR1/2) and c-Fos expression were analyzed immunohistochemically as reported^27,33^. d-Allulose (1 g kg^−1^) or saline (10 ml kg^−1^) was p.o. administered in male C57BL/6J wild-type mice and male *Glp1r* KO mice fasted 16 h. At 30 min (for pERK1/2 staining) or 90 min (for c-Fos staining) after injection, mice were transcardially perfused with phosphate buffer including 4% paraformaldehyde and 0.2% picric acid under anesthesia. The nodose ganglions (NGs) and brains were collected, postfixed in the same fixative for 2 h to overnight at 4 °C, and incubated in phosphate buffer containing 30% sucrose for 48 h. Longitudinal sections (8 µm) of NGs were cut with 48 µm intervals using a precision cryostat (Leica Microsystems, IL). Coronal sections (40 µm) of hindbrain were cut with 120 µm intervals using a freezing microtome. Rabbit polyclonal antibody to phospho-p44/42 MAPK (Thr202/Tyr204, pERK1/2) (1/500; #9101; Cell Signaling Technology, MA) and Alexa 488-conjugated goat anti-rabbit IgG (1:500; A11008; Life technologies, MD) were used. Fluorescence images were acquired with BX50 microscope and DP50 digital camera (Olympus, Tokyo, Japan). In c-Fos staining, anti-c-Fos antisera (sc-52, 1:10,000, Santa Cruz biotechnology, CA) were used as the primary antibody. Color was developed with nickel-diaminobenzidine (DAB). Neurons immunoreactive to pERK1/2 and c-Fos in medial NTS (bregma −7.32 to −7.76 mm) were counted.

### Subdiaphragmatic and hepatic-selective vagotomy

Bilateral subdiaphragmatic vagotomy was performed as previously reported^27^. A midline incision was made to provide wide exposure of the upper abdominal organ in male C57BL/6J mice anesthetized with tribromoethanol (200 mg kg^−1^, i.p.). The bilateral subdiaphragmatic trunks of vagal nerves along the esophagus were exposed and cut. In the sham operation group, these vagal trunks were exposed but not cut. The vagotomized and sham-operated mice were maintained on a nutritionally complete liquid diet for human baby (Chilmil, Morinaga, Tokyo, Japan). One to two weeks after the operation, feeding experiments and GTTs were performed. After experiments, to confirm whether the surgical vagotomy was successful, an increase in stomach weight, a reported phenotype of subdiaphragmatic vagotomy^55^, was checked.

Selective hepatic vagotomy was performed as described^56^. The ventral subdiaphragmatic vagal trunk was exposed as described above under anesthesia (200 mg kg^−1^ i.p. tribromoethanol). Since vagal hepatic branch forms a neurovascular bundle, this branch was selectively ligated by silk sutures and cut using microscissors. In the sham operation group, the hepatic branch was exposed but not cut. Hepatic vagotomized and sham-operated mice were recovered under chow (CE-2) diet for 1 week before experiments. It was reported that the hepatic vagotomy is judged successful if fasting-induced fat utilization in the epididymal fat tissue is decreased^57^. Hence, at the end of various measurements, mice were fasted overnight (16 h) and sacrificed and the epididymal fat weight was measured. We found an increased epididymal fat mass after hepatic vagotomy (sham 0.127 ± 0.010 g (*n* = 11) vs. hepatic vagotomy 0.190 ± 0.015 g (*n* = 12), *p* < 0.01 by unpaired *t*-test), confirming that the surgical hepatic vagotomy was successful.

### Measurements of [Ca^2+^]_*i*_ in single nodose ganglion neurons

Single neurons of nodose ganglia (NGs) were prepared from male C57BL/6J mice (6-weeks-old) as described^33,58^. NGs were excised from the mice anesthetized with α-chloralose and urethane (0.1 and 1 g kg^−1^, i.p.). The NGs were treated 20 min at 37 °C with 0.1–0.5 mg ml^−1^ collagenase Ia (Sigma), 0.4–0.6 mg ml^−1^ dispase II (Roche, Basel, Swiss), 15 µg ml^−1^ DNase II type IV (Sigma), and 0.75 mg ml^−1^ bovine serum albumin (Sigma) in HKRB supplemented with 5.6 glucose. Separated single neurons were cultured in Eagle’s MEM with 10% fetal bovine serum supplemented with 5.6 mM glucose for 12–24 h.

Measurements of [Ca^2+^]_*i*_ in the primary cultured single NG neurons were carried out as described previously^33,58^. Following incubating with 2 µM fura-2 AM (DOJINDO, Kumamoto, Japan) for 30 min at 37 °C, the cells were mounted in chamber and superfused at 1.3 ml min^−1^ at 30 °C with HKRB containing 5.6 mM glucose. Fluorescence ratio images at 510 nm following excitation at 340 and 380 nm were produced by an Aquacosmos ver. 2.6 (Hamamatsu Photonics, Shizuoka, Japan). When [Ca^2+^]_*i*_ changed within 5 min after addition of agents and their amplitudes were at least twice larger than the fluctuations of the baseline, they were considered responses. Only the neurons that responded to high KCl (55 mM) were analyzed.

### Construction of shRNAs and viral vector production

A target sequence for rat *Glp1r* was chosen to design short hairpin RNAs (shRNAs) (Accession No. NM_012728.1: 5′-GTATCTCTACGAGGACGAG-3′. 880 to 898). A scrambled oligonucleotide sequence was used for specificity control (5′- TTCTCCGAACGTGTCACGT-3′). To construct shRNAs, forward oligonucleotides were designed to contain the sense and antisense sequences connected with a hairpin loop (5′-CTGTGAAGCCACAGATGGG-3′) followed by a poly(dT) termination signal. The annealed forward and reverse oligonucleotides were ligated into the pAAV-U6-ZsGreen1 vector (Takara Bio, Japan). Using this vector, the shRNA expression was driven by the mouse U6 promoter, whereas green fluorescence protein, ZsGreen1 expression was controlled by the cytomegalovirus (CMV) promoter. Briefly, AAV9-*Glp1r*-shRNA and AAV9-Scr-shRNA viruses were produced following triple-transfection of HEK293 cells with pAAV-shRNA, an adenoviral helper plasmid pAdeno, and a chimeric helper plasmid encoding AAV2 rep/AAV9 cap genes (pAAV2rep/AAV9cap) (a gift from Dr. James M Wilson). The rAAV particles were purified by a dual ion-exchange procedure using high-performance membrane absorption as previously described^59^. At 72 h after transfection, cell pellets were resuspended in 30 ml of Tris-buffered saline (TBS: 100 mM Tris-HCl [pH 8.0], 150 mM NaCl). Recombinant AAV was harvested by five cycles of freeze–thawing of the resuspended pellet. The crude viral lysate was initially concentrated by a brief two-tier CsCl gradient centrifugation for 3 h and then the vector fraction was dialyzed in MHA buffer (3.3 mM morpholinoethanesulfonic acid [MES], 3.3 mM HEPES [pH 7.5], 3.3 mM sodium acetate). Chromatography was performed with an AKTAexplorer 10 S fast protein liquid chromatography (FPLC) system (GE Healthcare Life Sciences). First, a dialyzed vector-containing fraction was loaded onto a cation-exchange membrane (Acrodisc unit with Mustang S membrane; Pall, East Hills, NY). The flow-through sample was further loaded onto an anion-exchange membrane (Acrodisc unit with Mustang Q membrane; Pall). After washing, bound virus on the Mustang Q membrane was eluted with a 0–2 M linear NaCl gradient in MHA buffer. Recombinant rAAV particle number was determined by quantitative real-time PCR. Primers sequences were 5′-ACCGTGTACAAGGCCAAGTC-3′ and 5′-GTCAGCTTGTGCTGGATGAA-3′.

### AAV-mediated shRNA interference of nodose ganglion in rats

The left NG of male Wistar rats was surgically exposed under anesthesia (Tribromoethanol 200 mg kg^−1^ i.p.). A glass capillary (30–50 µm tip) was used to administer 2 µL AAV solution (Control vs. *Glp1r* knockdown (KD), 1.0 × 10^12^ vector genomes ml^−1^ with 0.05% Fast Green) into the left NG with a BJ-110 microinjector (BEX, Tokyo, Japan). At the operation, the success of microinjection was determined by Fast Green Dye filling in the NG. We allowed animals to recover for at least 2 weeks to ensure expression of the viral constructs.

On experimental day at 9:50, d-allulose (1 g kg^−1^, 10 ml kg^−1^) or saline (10 ml kg^−1^) was p.o. administered into the rats fasted 16 h, and at 10:00 standard chow was given and food intake was measured.

After feeding experiments, the left and right NGs were isolated from the rats and the AAV-mediating ZsGreen1 expression in NGs was observed under fluorescence microscope. Thereafter, these NGs and the hypothalamus from the rats were immediately soaked in RNAlater (Thermo Fisher Scientific). Total RNA was extracted using Trizol (Thermo Fisher Scientific) and cDNA were synthesized by Verso cDNA synthesis kit (Thermo Fisher Scientific). Quantitative real-time PCR was performed using SYBR Green (SYBR Select Master Mix, Applied Biosystems) on a ViiA7 system (Applied Biosystems), and results were analyzed using the 2ddCt methods. Receptor mRNA expression (*Glp1r* and *Cckar*) was normalized to 36b4 mRNA levels. Primers sequences were 5′-ATCAAAGACGCTGCCCTCAA-3′ and 5′-CCACGCAGTATTGCATGAGC-3′ (*Glp1r*); 5′-GCTTTGAAGGTCATCGCTGC-3′ and 5′- AGCAGGAATGTTTGCCAGGA-3′ (*Cckar*); 5′-TGTTGAACATCTCCCCCTTCT-3′ and 5′-GACACCCTCTAGGAAGCGAG-3′ (*36b4*).

### Statistical analysis

We did not perform power calculations, but the sample size and animal number for each group were chosen based on study feasibility and prior knowledge of statistical power from previously published experiments. Animals were randomly allocated to individual experimental groups and were performed without blinding. All data were expressed as means ± SEM. All groups from our data showed normal variance, and statistical analysis was performed by two-tailed unpaired *t*-test or by one-way or two-way ANOVA. When ANOVA indicated a significant difference among groups, these groups were compared by Dunnett’s, Tukey’s or Bonferroni’s post hoc test. All statistical analyses were performed using Prism 5 (GraphPad Software, CA). *p* < 0.05 was considered significant.

### Data availability

The data that support the findings of this study are available from the corresponding authors upon reasonable request.

## Electronic supplementary material

Supplementary Information
