# Supplementary material for: GLP-1 release and vagal afferent activation mediate the beneficial metabolic and chronotherapeutic effects of D-allulose
Source: Nat Commun. 2018 Jan 9;9:113. doi: 10.1038/s41467-017-02488-y (PMC5760716; doi:10.1038/s41467-017-02488-y)
Supplement: Supplementary file 1 — Supplementary Information [file 41467_2017_2488_MOESM1_ESM.pdf]

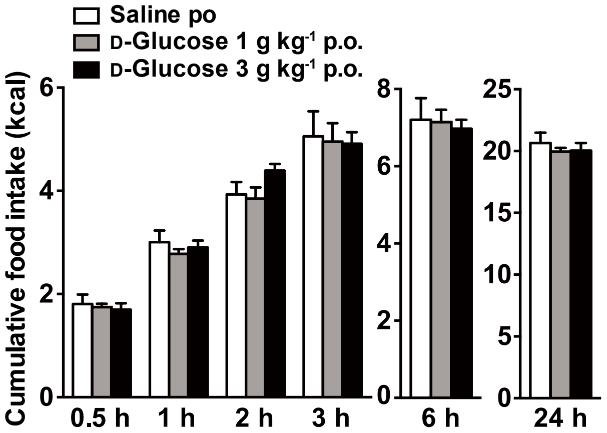

Supplementary Figure 1

Peroral administration of D-glucose does not alter food intake in C57BL/6J mice. Peroral (p.o.) administration of 1 and 3 g kg^{-1} D-glucose did not alter cumulative food intake in C57BL/6J mice fasted for 16 h. One and 3 g kg^{-1} D-glucose had 0.0926 ± 0.0013 and 0.2740 ± 0.0038 kcal, respectively, which were included in the cumulative food intake. n = 5. Error bars are SEM

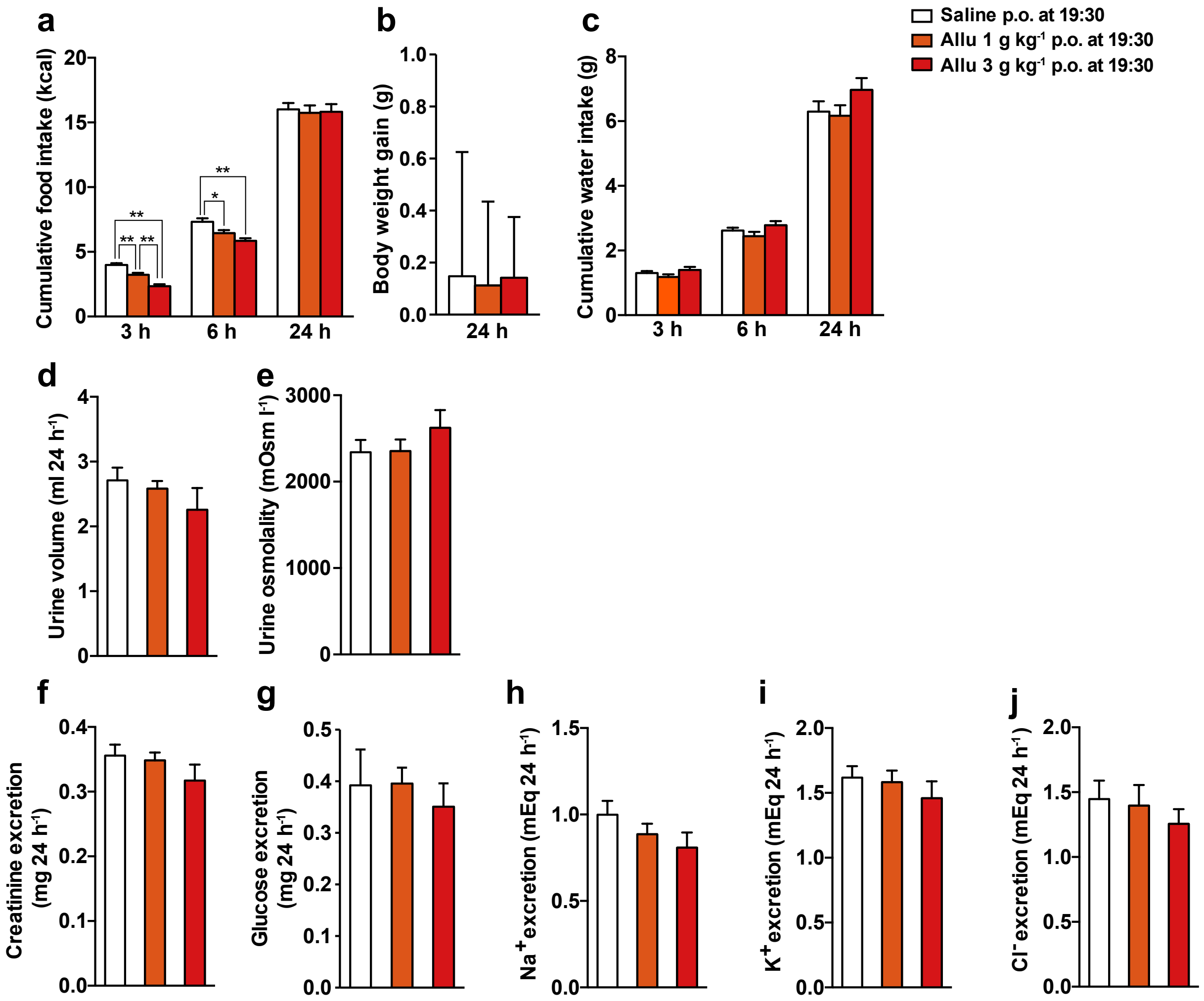

Supplementary Figure 2

Single p.o. administration of D-allulose (Allu) decreases food intake without altering water intake, urine volume and osmolality, and urinary excretion of creatinine, glucose and electrolytes. (**a-c**) Cumulative food intake (**a**), body weight gain (**b**) and cumulative water intake (**c**) after p.o. administration of saline or Allu (1 or 3 g kg^{-1}) at 19:30 in normal C57BL/6J mice housed single home cages. n = 11-12 (**a, c**) and 5 (**b**). (**d-j**) Urine was collected for 24 h after p.o, administration of saline or Allu (1 or 3 g kg^{-1}) at 19:30 in normal C57BL/6J mice housed individual metabolic cages were collected, followed by measurements of urine volume, osmolality, creatinine, glucose and electrolyte (Na^+, K^+, Cl^-) concentrations. Error bars are SEM

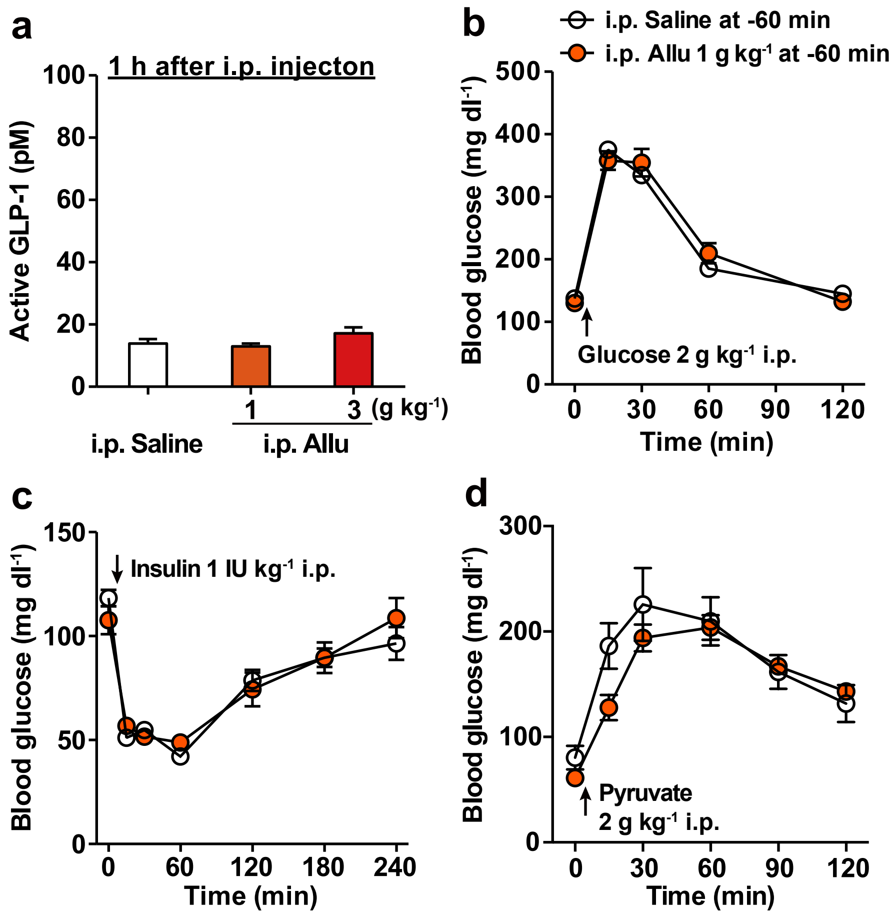

Supplementary Figure 3

I.p. administration of D-allulose has no effects on plasma GLP-1 levels, glucose tolerance, insulin action and hepatic glucose production. (**a**) I.p. injection of 1 and 3 g kg^{-1} Allu did not alter plasma active GLP-1 levels in mouse portal vein at 1 h after administration. n = 6. (**b-d**) Allu, i.p. administered 1 h prior to ipGTT (**b**), ITT (**c**) and PTT (**d**), did not alter blood glucose profiles in these tests in mice. n = 5-6. Error bars are SEM

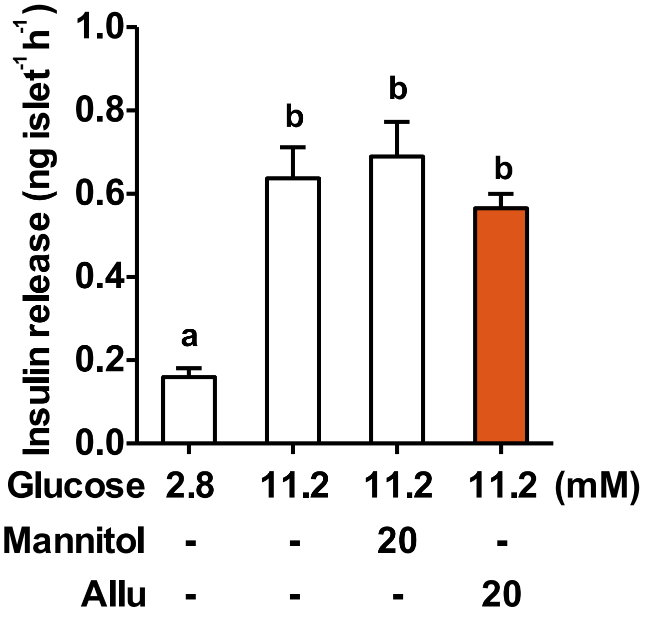

Supplementary Figure 4

D-Allulose dose not stimulate insulin secretion from isolated islets *ex vivo*. Effect of Allu on insulin secretion from isolated mouse islets in static incubation. Rise in glucose concentration (11.2 mM) stimulated insulin secretion from islets. The glucose-stimulated insulin secretion was altered neither by 20 mM Allu nor by its osmolality control mannitol. n = 9. Different letters indicate $P < 0.05$ by one-way ANOVA followed by Tukey's test. Error bars are SEM

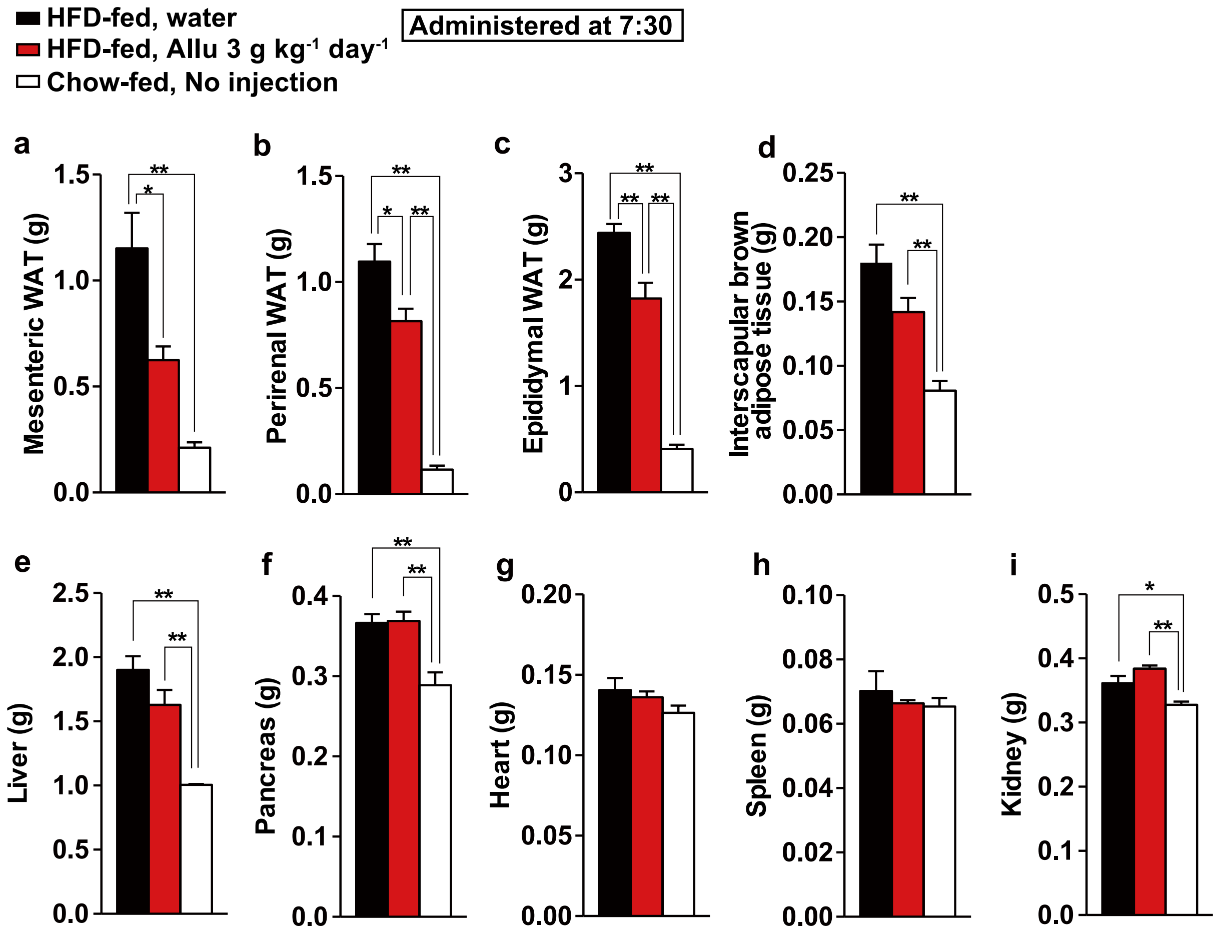

Supplementary Figure 5

Subchronic p.o. administration of 3 g kg^{-1} D-allulose at LP onset significantly reduces mesenteric, perirenal and epididymal adipose tissue weights but not those of principal peripheral organs. Weights of organs in HFD-fed mice at Day 11 following treatment with p.o. Allu (3 g kg^{-1} day^{-1}) or water (10 ml kg^{-1} day^{-1}) once daily for 10 days and in control mice fed standard chow (Chow-fed). **P* < 0.05, ***P* < 0.01 by one-way ANOVA followed by Tukey's test. n = 5-6. Error bars are SEM

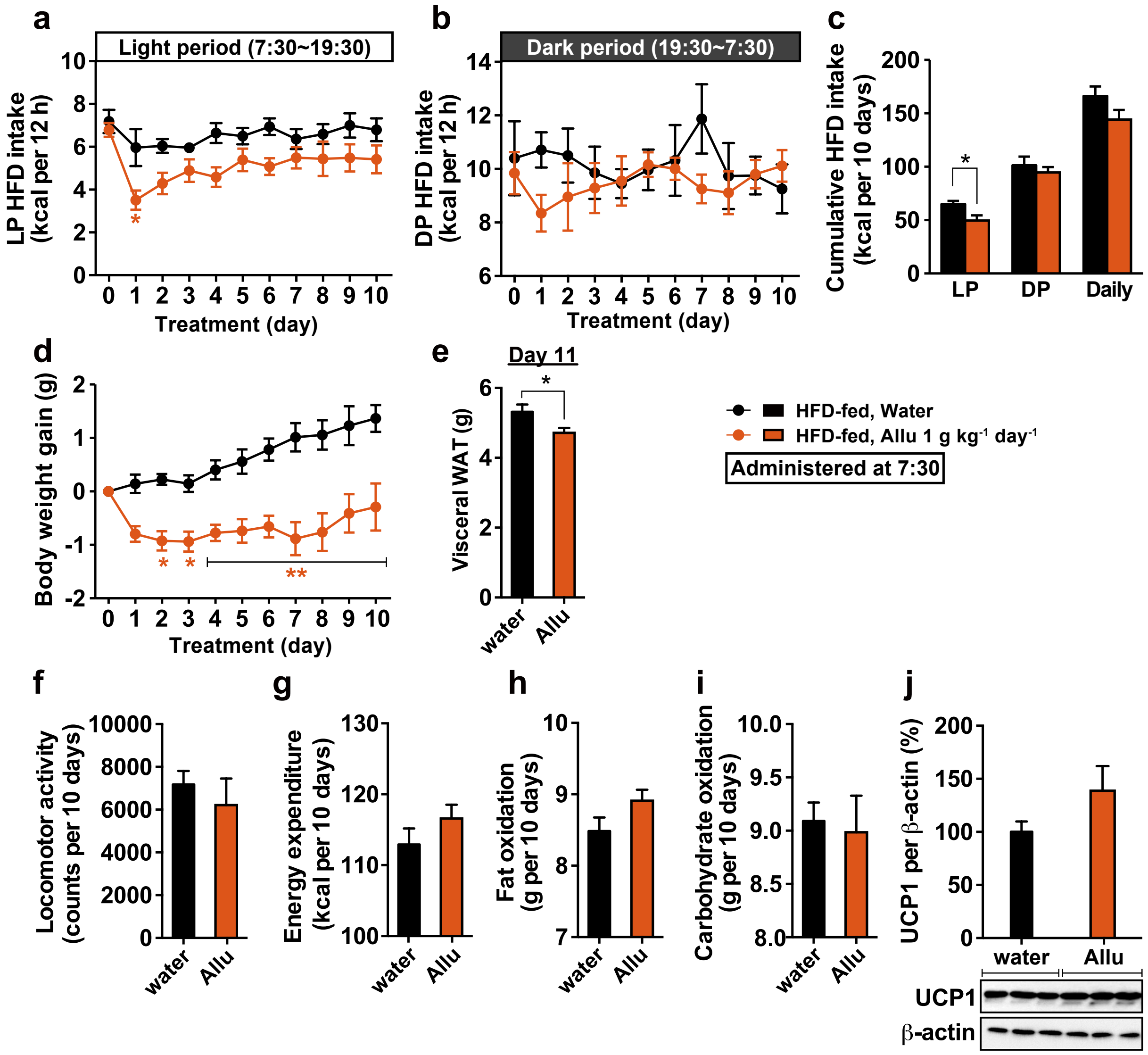

Supplementary Figure 6

Subchronic p.o. treatment with 1 g kg^{-1} day^{-1} D-allulose at LP onset ameliorates LP hyperphagia and visceral obesity, with tendency of modest increases in energy expenditure and UCP1 expression in HFD-fed obese mice. In experiments with the same protocol as figure 6 but in individual small acryl calorimeter chambers, respiratory gases for 10 days were measured to assess energy expenditure. Daily p.o. administration of 1 g kg^{-1} day^{-1} Allu at LP onset (7:30) consecutively suppressed LP HFD intake (**a**) without altering DP HFD intake (**b**), thereby significantly decreasing cumulative LP HFD intake but not DP and daily HFD intake (**c**). Allu treatment significantly attenuated body weight gain (**d**) and weight of visceral WAT including mesenteric, perirenal and epididymal WAT at Day 11 (**e**). Allu treatment for 10 days tended to increase energy expenditure (**g**), fat oxidation (**h**) and UCP-1 expression in interscapular brown adipose tissue (**j**) without changing locomotor activity (**f**) and carbohydrate oxidation (**i**). In (**j**), the detected bands specific for UCP1 and β-actin were 32 and 42 kDa, respectively. n = 5-6. *P < 0.05 and **P < 0.01 by two-way ANOVA followed by Bonferroni's test (**a, d**). *P < 0.05 by unpaired t-test (**c, e**). Error bars are SEM

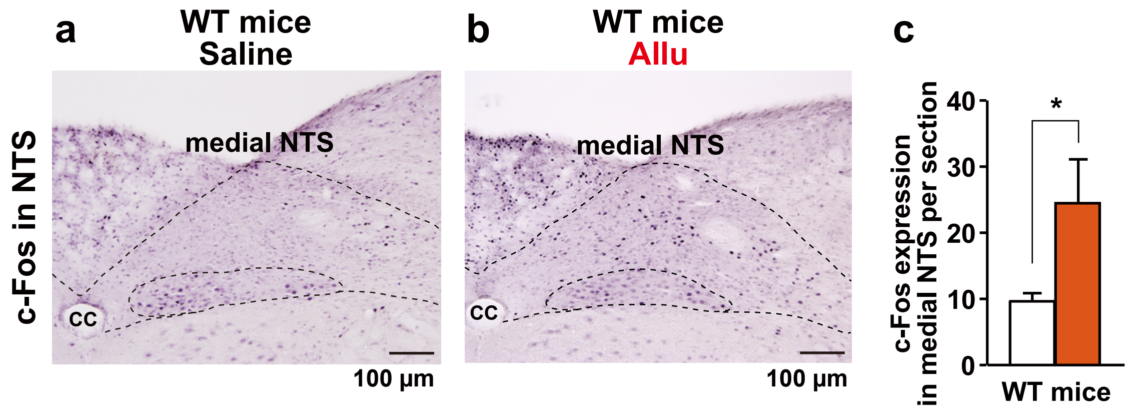

Supplementary Figure 7

Peroral D-allulose induces c-Fos expression in medial NTS. (**a-c**) Allu (1 g kg^{-1}, p.o.) increased c-Fos expression in medial NTS in C57BL/6J mice. n = 7-6. *$P < 0.05$ by unpaired t-test. Scale bar, 100 µm. Error bars are SEM

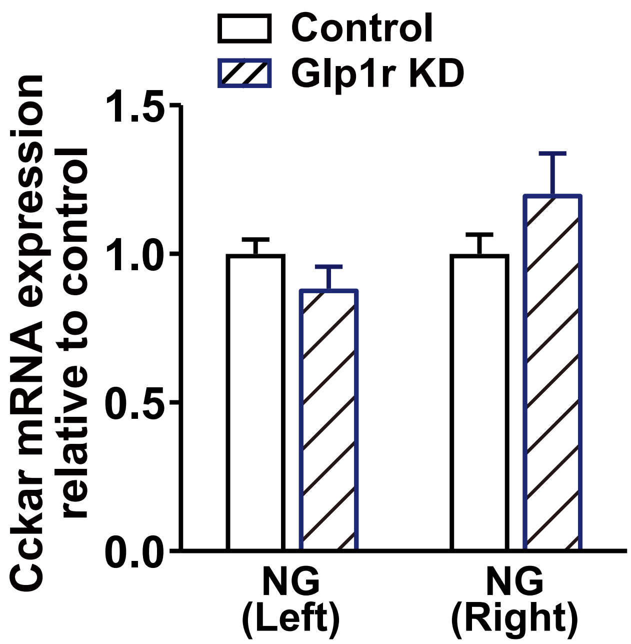

Supplementary Figure 8

***Glp1r* knockdown (KD) in left NG did not change expression of CCK-A receptor mRNA in left and right NGs.** Relative expression of CCK-A receptor (Cckar) mRNA in the left NG (AAV injected) and right NG (not injected) in *Glp1r* KD and control rats. n = 11. No significant difference between *Glp1r* KD and control by unpaired t-test. Error bars are SEM

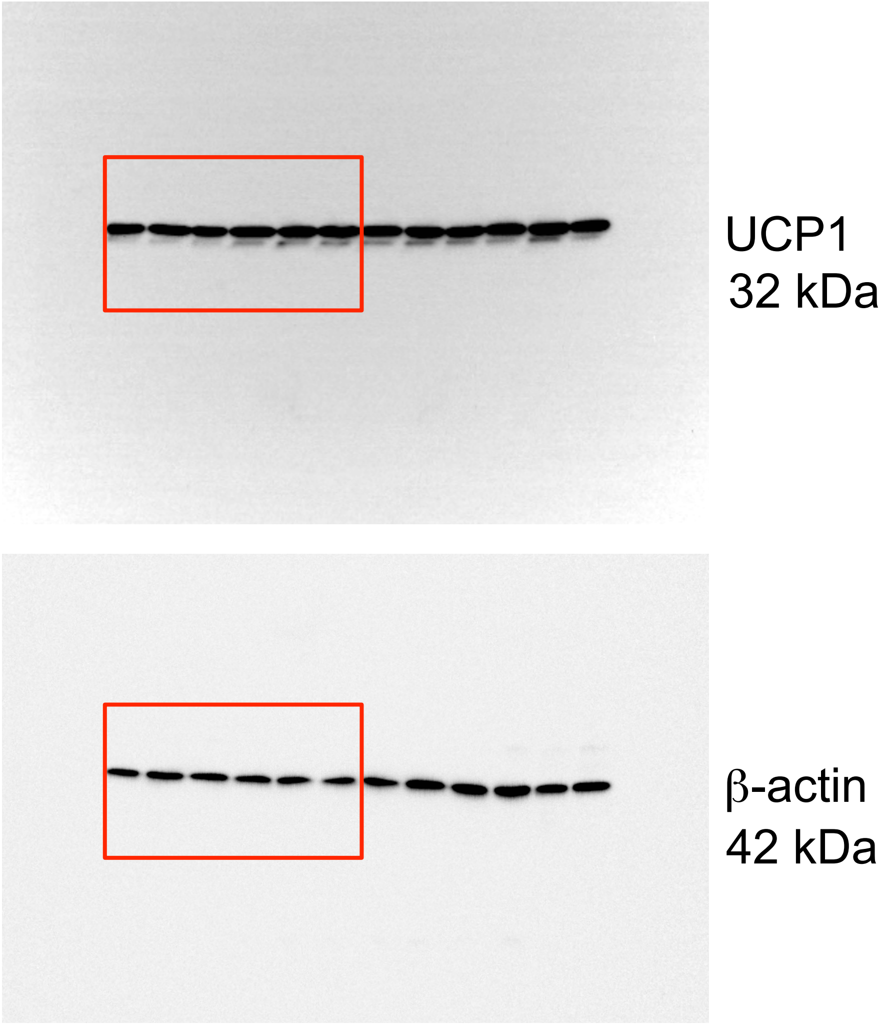

Supplementary Figure 9
Uncropped full-length image of western blot in Supplementary Figure 6j.
Each band size was estimated using dual color standards (#161-0374, BIO-RAD).
